# Cell heterogeneity contributes to the variable response of HIV-1 to latency-reversing agents

**DOI:** 10.1126/sciadv.aeb1435

**Published:** 2026-10-09

**Authors:** Rachel Topno, Hussein Karaki, Flavia Mazzarda, Oriane Pourcelot, Manon Philippe, Kazem Zibara, Ovidiu Radulescu, Edouard Bertrand

**Affiliations:** ^1^ IGH, University of Montpellier and CNRS, 141 Rue de la Cardonille, Montpellier 34094, France.; ^2^ LPHI, University of Montpellier, CNRS and INSERM, Place Eugene Bataillon, Montpellier 34095, France.; ^3^ Research and Analysis Platform in Environmental Sciences (EDST-PRASE), Lebanese University, Hadath P.O. Box 14-6573, Lebanon.

## Abstract

Transcriptional noise contributes to gene expression variability, but its origin and impact in HIV-1 latency remain incompletely characterized. Here, we combine a dual-copy MS2-tagging system with dedicated mathematical analysis to investigate the variability of HIV-1 transcription in live cells. In the basal state, the transcriptional activity of proviruses located in the same cell was uncorrelated, indicating that expression variability primarily comes from the intrinsic stochasticity of promoter dynamics. Upon stimulation with diverse latency-reversing agents, transcription of viral copies became more correlated within cells than across them, revealing a shift from promoter-driven to cell state–driven variability. Analysis of the molecular drug targets confirmed variable effects across cells. Our findings indicate that cellular heterogeneity can shape the response to latency-reversing agents and demonstrate how quantitative tools can dissect noise sources. This work offers mechanistic insights into HIV-1 latency and informs strategies to target the latent viral reservoir.

## INTRODUCTION

Live-cell imaging studies have shown that transcription does not proceed at a uniform rate even under steady-state conditions. Instead, it occurs in bursts, during which many mRNAs are rapidly transcribed, followed by periods of transcriptional silence (*1*). This burst-like behavior reflects the stochastic nature of promoter activity, with variable durations of active and inactive periods, an effect commonly referred to as transcriptional noise [reviewed in (*1*–*5*)]. In a population of clonal cells exhibiting bursting behavior, this noise manifests as heterogeneity: Some cells appear transcriptionally active, while others remain silent. This heterogeneity can have important phenotypic consequences [see (*2*–*5*) and therein]. Understanding the various sources of transcriptional noise is a fundamental but yet incompletely resolved question (*6*).

Transcriptional noise has two major sources: extrinsic and intrinsic, which are usually distinguished by considering the behavior of identical copies of a gene within a cell (*7*–*9*). Intrinsic noise appears to be generated by the stochasticity of molecular events arising at a single transcription site (TS), such as transcription factors binding-unbinding, nuclear architecture modifications (DNA looping, promoter-enhancer interaction, and nuclear compartmentalization), local chromatin changes (nucleosome occupancy, histone modifications, and occupancy of regulatory elements), polymerase pausing, and DNA supercoiling (*10*, *11*). Intrinsic noise affects various gene copies within a cell in an independent, uncorrelated manner (*6*) and it is generally modeled as a promoter transitioning stochastically between transcriptionally active and inactive states (*12*). Single-molecule studies such as single-molecule DNA footprinting or electron microscopy were able to reveal and characterize these molecular states (*13*, *14*).

In contrast to intrinsic noise, extrinsic noise affects the copies of a gene within the same cell in a correlated manner. It can result from cell-to-cell fluctuations in the concentration of transcription factors or molecules involved in signal transduction, heterogeneity in the metabolic cellular status, cell-cycle stage, or in the cellular environment (*15*–*18*). Identifying and separating intrinsic and extrinsic noise is crucial because they account for distinct types and sources of variability; considering intrinsic noise alone therefore provides an incomplete and potentially misleading model of the observed fluctuations (*19*). Clarifying the contributions of extrinsic and intrinsic sources of noise is also particularly important in biological systems where transcriptional noise has phenotypic consequences. This is the case of HIV-1 [(*18*, *20*) for review], where stochastic variation of promoter activity has been proposed to control latency entry and exit, as well as the heterogeneous response of cells to latency-reversing agents (LRAs), which limits current curing strategies (*21*, *22*).

The HIV-1 genome consists of a single transcription unit that is integrated into cellular chromatin. Transcription is carried out by the host cellular machinery, resulting in primary transcripts that remain unspliced to be packaged into virions, or that are alternatively spliced to produce the various viral mRNAs and proteins. The HIV-1 promoter contains binding sites for a number of transcription factors including nuclear factor κB (NF-κB), SP1, and the TATA-box binding protein (TBP). NF-κB activates viral transcription in response to extracellular signals such as the tumor necrosis factor–α (TNFα) cytokine, while SP1 is required for the basal and activated transcriptional activity of the virus (*23*–*27*). The virus also encodes the Tat protein, which stimulates viral transcription by more than 100-fold, thus triggering a positive feedback loop. Tat binds to the nascent TAR RNA together with P-TEFb, which enables the release of RNA polymerases from a promoter proximal pause (*28*, *29*). Critically, the HIV-1 virus can exist in a latent and an acute state, which are mainly controlled at the transcriptional level. In latent cells, viral transcription is minimal and the Tat feedback loop is OFF. In acutely infected cells, viral transcription is active, producing high levels of Tat and fully activating the viral promoter. The discovery of transcriptional noise has generated considerable interest in HIV-1 biology, particularly in relation to its role in latency entry and exit. Early studies using green fluorescent protein (GFP)–tagged mini-viruses suggested that these viruses behave as bi-stable transcriptional switches, which stochastically alternate between meta-stable transcriptionally active (ON) and silent (OFF) states resembling acute and latent states, respectively (*30*–*34*). Direct imaging of HIV-1 transcription using MS2-tagged reporters in living cells further showed that the viral promoter stochastically fluctuates between active and inactive states independently of Tat, as a result of gene bursting (*35*, *36*). In absence of Tat, this bursting could potentially trigger spontaneous latency exit by inducing the Tat feedback loop.

In patients, HIV infection can be controlled with highly active antiretroviral therapy. However, interruption of treatment leads to viral rebound within weeks and lifelong treatment is required, posing risk of secondary drug effects and the emergence of resistant strains (*37*). The viral rebounds are primarily driven by latently infected cells, which form the viral reservoir. This reservoir is established early in infection and may be maintained by low-level viral propagation, even under antiretroviral therapy (*38*). The half-life of the reservoir is estimated to be around 44 months, meaning its eradication could require more than 60 years of continuous treatment (*21*, *22*, *37*). This highlights the need for new strategies that target the reservoir for the development of an effective cure. One approach is the “shock and kill” strategy (*39*) whose goal is to reactivate viral transcription using LRAs, thereby allowing the immune system to recognize and eliminate latent cells. Unfortunately, this approach is currently limited by the inability to activate all latent viruses, thus hindering their complete eradication [see (*40*) and therein]. This inability is due to the stochastic response of latent viruses to LRAs, with some viruses activating transcription and others not, even in clonal populations (*4*, *41*). This variability has been observed in model systems and also directly demonstrated in patient cells (*20*, *42*).

While stochastic viral responses are a key limitation for therapies targeting the reservoir, their origins remain only partially understood. In particular, it is unclear to what extent this variability arises from intrinsic versus extrinsic sources of noise. Intrinsic noise reflects stochastic fluctuations in the molecular dynamics of latent viral promoters, causing individual proviruses to switch over time between transcriptionally permissive and nonpermissive states, thereby generating heterogeneous reactivation probabilities even in otherwise identical cells. In contrast, extrinsic noise arises from cell-to-cell differences in transcription factor availability, signaling activity, metabolic state, or cellular microenvironment. Distinguishing these sources of variability is therefore essential, as they have different biological and therapeutic implications: Intrinsic noise implies fundamentally probabilistic reactivation dynamics at the single-cell level, whereas extrinsic noise suggests that subsets of cells may be systematically more or less permissive to reactivation. More generally, understanding how these noise sources interact with latency would provide important insight into the mechanisms governing reservoir persistence and treatment response in patients.

Here, we developed a system to image in real time the transcription of two identical viral copies integrated into human cells. We quantitatively dissected the respective contributions of intrinsic and extrinsic sources of transcriptional heterogeneity under basal conditions and upon stimulation with diverse LRA treatments. We show that the heterogeneity in basal viral transcription is entirely driven by intrinsic noise, related to promoters being in different molecular states, while the heterogeneity in the response to LRAs shows a large contribution of extrinsic factors related to the variable properties of individual cells. Cellular heterogeneity is thus an important issue for therapeutic approaches targeting the viral reservoir, and likely plays an important role in the reservoir dynamics in patients.

## RESULTS

### Generation of a two-copy HIV-1 system to measure the contribution of extrinsic and intrinsic sources of noise

Inserting multiple copies of a reporter gene into the same cell and measuring the correlation of their expression provides a robust way to quantify the relative contributions of intrinsic and extrinsic noise (*9*). To construct such a system, we generated two identical HIV-1 vectors bearing different selection markers and introduced them at a precise location in the human genome by CRISPR-Cas9–mediated homologous recombination. We chose an integration site in the antisense direction of the TBC1D5 gene, which was previously identified as a viral integration site in a patient (*43*), and the viral vector that we integrated was similar to the one we used previously to image HIV-1 transcription (*35*, *36*) (Fig. 1A). This vector harbored the 5′ long terminal repeat (LTR) with the viral promoter and TAR element, the splicing donor SD1, splice acceptor A7 and the 3′ LTR with the 3′-end processing and polyadenylation site. We used the MS2/MCP system to image viral RNA in living cells (*44*) and inserted 128 MS2 loops in the intron between SD1 and A7 (*35*). We previously showed that this intron splices post-transcriptionally and enables imaging transcription independently from splicing (*36*). The 128xMS2 tag also enables the visualization of single RNAs for extended time periods and we used the newly developed MCP-tdStayGold variant to further improve RNA imaging (*45*). Overall, this system allows visualization of single RNAs with exceptional photostability and brightness, ensuring the detection of every transcriptional event over long time periods. The viral vector did not express Tat and thus corresponded to a transcriptional status analogous to the one of latent cells.

**Fig. 1.**
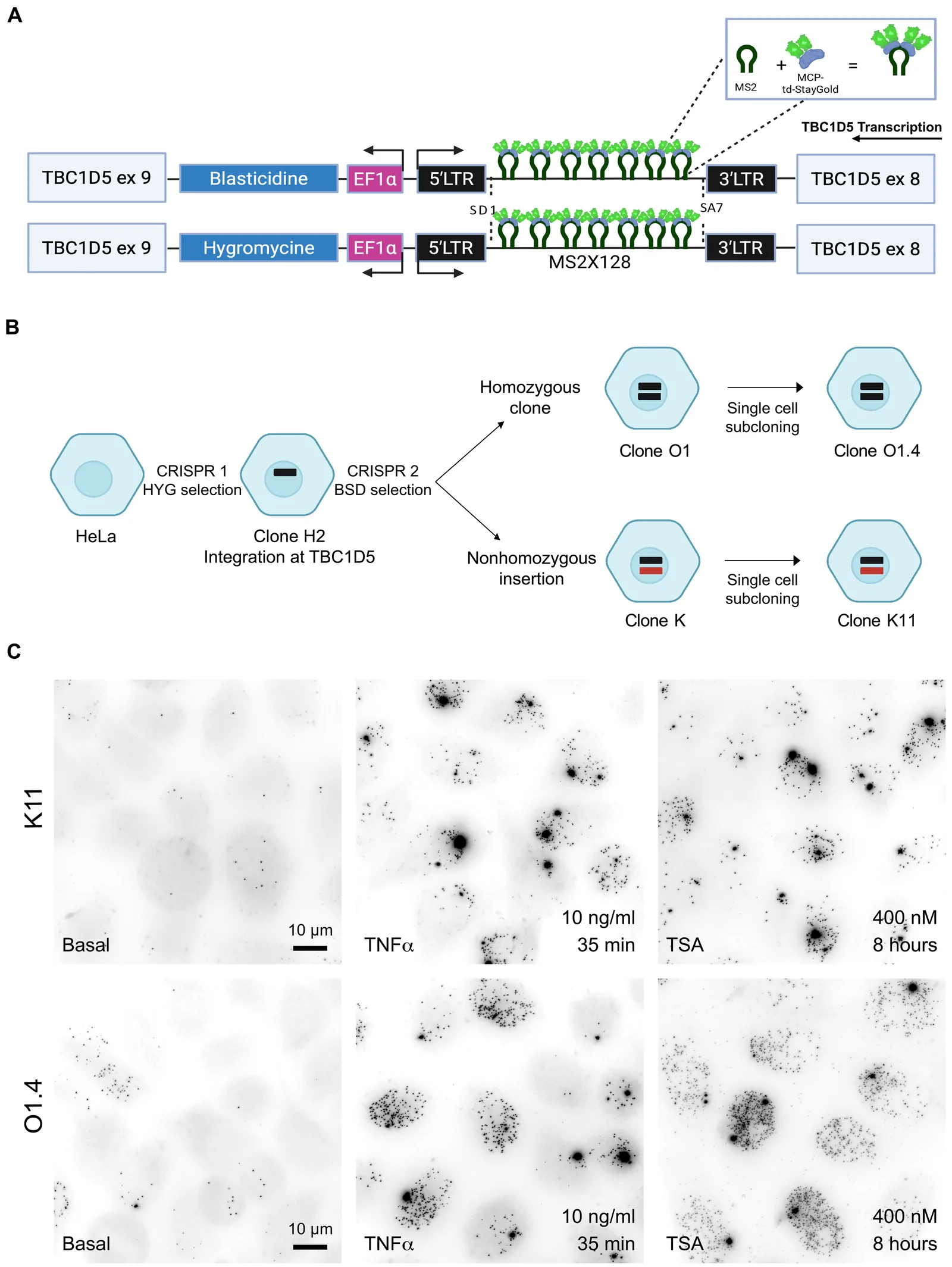
Generation and characterization of two-copy HIV-1 CRISPR clones. (**A**) Schematic representation of two CRISPR repair cassettes designed to insert an HIV-1 reporter containing 128 MS2 stem-loops in the TBC1D5 gene in the antisense oriantation. Key components of the reporter include the major HIV-1 splice donor site (SD1), the last HIV-1 splice acceptor site (SA7), homology arms targeting TBC1D5 (TBC1D5 HA), and the selection markers blasticidin and hygromycin. The human elongation factor 1 alpha (EF1α) promoter drives expression of the selectable marker. The MS2 coat protein (MCP-tdStayGold; green) binds to the MS2 stem-loops. (**B**) Schematic outlining the experimental workflow for generating the CRISPR-edited HeLa clones. Two rounds of CRISPR transfections and clone selection were performed. In the first round, a cassette with the hygromycin selection marker was used, resulting in the H2 clone. A second round of CRISPR transfection using the blasticidine marker was performed on the H2 clone, yielding two subclones: the O1 homozygous clone and the K clone with a nonhomologous integration site. Both clones were subsequently subcloned to produce the O1.4 and K11 clones, respectively. (**C**) The micrographs represent smiFISH microscopy images of clone K11 (top) and O1.4 (bottom) under basal, TNFα, and TSA stimulated conditions (from left to right). The concentration and duration of treatment are indicated in the bottom-right corner of each image.

The two copies of the MS2-tagged viral reporter were inserted in the genome sequentially using two rounds of CRISPR editing in HeLa cells (Fig. 1B). In the first round, we used the repair cassette bearing the hygromycin selectable maker, and after polymerase chain reaction genotyping different clones, we identified a correct integration in clone H2 (fig. S1B). After subcloning, this cell line was further modified by CRISPR with the second repair cassette carrying the blasticidin selection maker, and two clones were characterized in detail (Fig. 1B and fig. S1B). The first clone, referred to as O1.4, carried a correct integration for the second CRISPR and was thus homozygous with the two viral reporters integrated in the two alleles of the TBC1D5 gene. The other clone, K11, had the second repair cassette integrated in a different site, and was thus heterozygous with respect to the TBC1D5 gene. The K11 clone allowed us to evaluate the contribution of extrinsic noise independently of the integration site.

The two clones were subjected to single-cell sorting and subcloned to ensure homogeneity, and analyzed by fluorescent microscopy to assess the quality of RNA imaging. Viral transcription was activated and RNA expression was assessed with smiFISH using probes hybridizing to the MS2 sequences. We observed two bright spots corresponding to the two expected viral TSs, as well as many dimmer spots corresponding to single RNAs (fig. S1A). Both colocalized with signals from the MCP-tdStayGold, indicating the accuracy and sensitivity of the RNA imaging system (fig. S1A and Supplementary Movies).

### SmFISH indicates a lack of correlation between HIV-1 copies in the basal state and moderate correlation during promoter activation

To determine the transcriptional activity of the two integrated copies of the MS2-tagged reporter, we performed smiFISH under basal and activated conditions and quantified the resulting signals. Specifically, we used two well-known transcriptional inducers of HIV-1, TNFα and Trichostatin A (TSA; Fig. 1C). TNFα activates NF-kB, which directly binds the HIV-1 promoter (*46*–*48*), while TSA inhibits histone deacetylases and promotes the formation of an open chromatin (*49*). Under basal conditions, smiFISH revealed low levels of nascent RNAs from either allele, in both clones K11 and O1.4 (0.3 and 0.4 RNA per TS; Fig. 2), yielding an average of 2.7 and 4.2 molecules of released RNAs per cell, respectively (fig. S2). Following treatment with TNFα for 35 min and TSA for 8 hours, we observed a marked increase in transcriptional output, as evidenced by a higher number of nascent and released RNAs for both the K11 and O1.4 clones, and for both reporter copies in each clone (Fig. 2 and fig. S2). TNFα treatment resulted in a 25-fold increase in nascent RNAs for K11 and 22 fold in O1.4, and a 7 fold increase in released RNAs in K11 and 6 fold in case of O1.4. TSA led to a 36- and 12-fold increase in nascent RNA for K11 and O1.4, respectively, and a 13- and 14-fold increase in released RNAs. This was in line with the expected response to these transcriptional inducers (*26*, *50*–*52*). These results demonstrate that the CRISPR-integrated HIV-1 system is functionally responsive to known transcriptional inducers, validating the system’s utility for monitoring viral transcription in live cells.

**Fig. 2.**
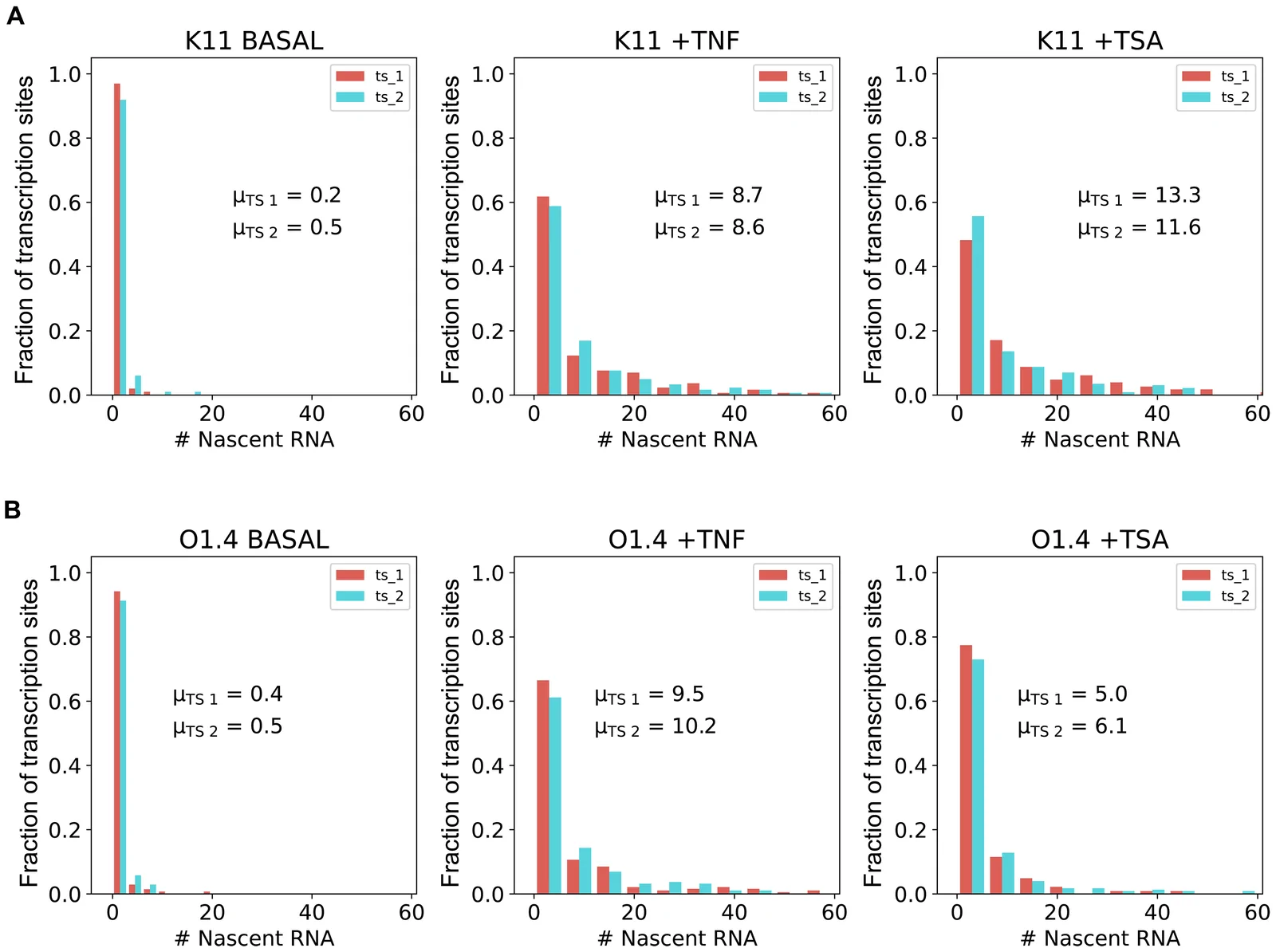
Transcriptional activity of the two HIV-1 copies under basal condition and during activation (**A** and **B**) The graphs display the distribution of nascent RNA counts per transcription site (TS) for the two reporter copies, distinguished by color (red and blue), for K11 (A) and O1.4 (B). The mean nascent RNA count for each transcription site (denoted as μ_TS1_ and μ_TS2_) is provided in the center of the plot. The *x* axis represents the number of nascent RNA counts, while the *y* axis shows the fraction of transcription sites exhibiting those counts. The number of cells in clone K11 used for basal, TNFα, and TSA treatments are 99, 301, and 228, respectively, while the number of cells used for Basal, TNFα and TSA treatments are 138, 188, and 226, respectively in clone O1.4.

Next, we compared the activities of the two HIV-1 copies located in the same cell. Scatter plots comparing the number of nascent RNAs for the two copies showed an absence of correlation in the basal state, for both K11 and O1.4 (Fig. 3, A and B). In contrast, a weak correlation was observed in the K11 clone 35 min after TNFα addition and a higher one was seen 8 hours after TSA addition (Pearson correlation coefficient of 0.15 and 0.26, respectively). For the O1.4 clone, a slightly negative correlation was seen after TNFα addition (see Discussion below for possible interpretations), and a positive one after TSA treatment. We further measured the total, extrinsic and intrinsic noise using the total law of variance (*9*). We found that the total noise levels decreased after transcriptional induction, for both K11 and O1.4 clones (Fig. 3C). Meanwhile, the proportion of extrinsic noise increased, reaching up to ∼20% in both clone K11 and O1.4 after TSA addition (Fig. 3, D and E).

**Fig. 3.**
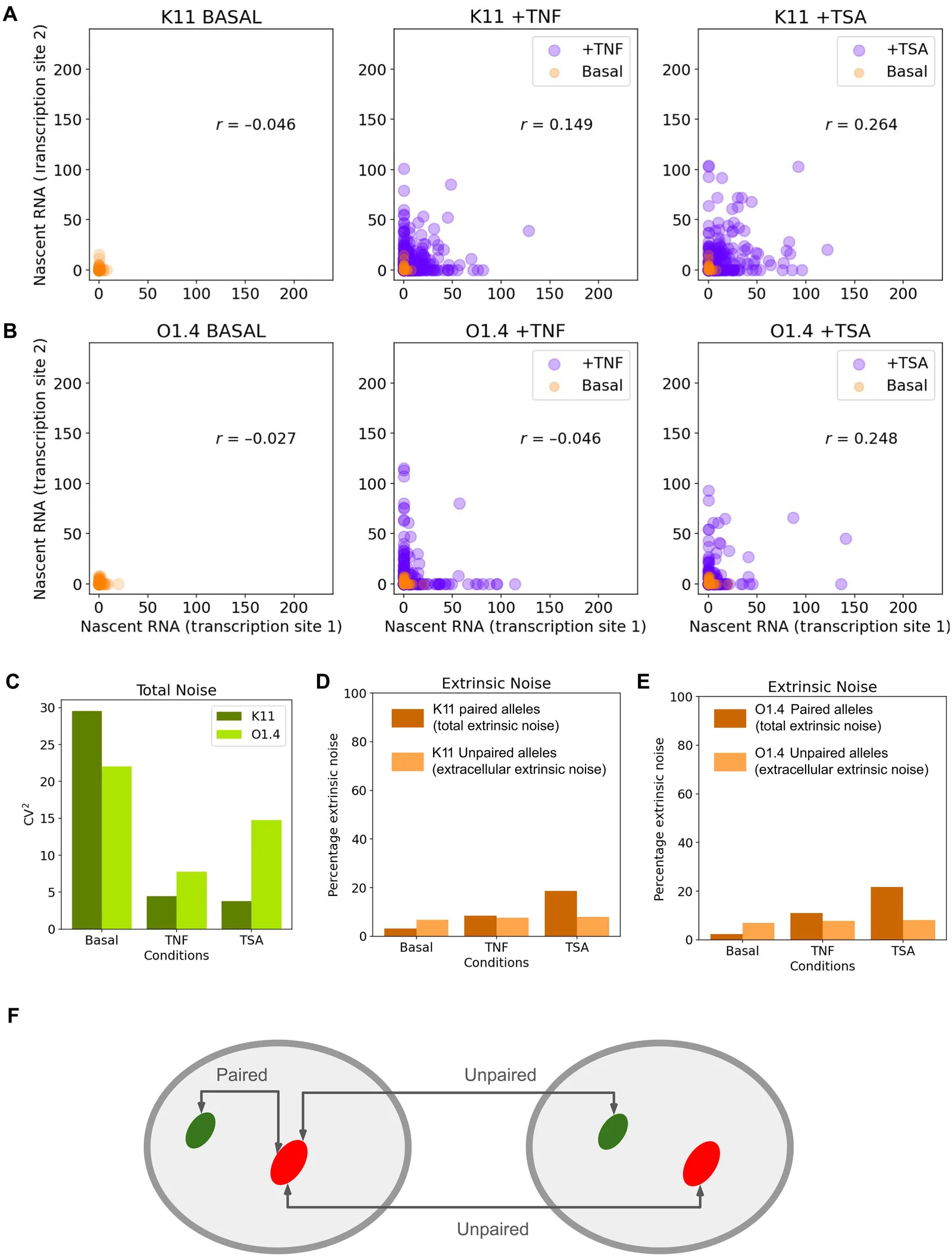
SmiFISH analysis reveals the presence of extrinsic noise and cellular heterogeneity. (**A** and **B**) Correlation scatter plots for nascent RNA counts from transcription site 1 (*x* axis) and transcription site 2 (*y* axis) for clones K11 and O1.4, respectively. The Pearson correlation coefficient (*r*) is displayed in the center of each plot. From left to right, the plots represent the basal, TNFα, and TSA treatment conditions. (**C**) Bar plot showing the noise estimates (CV^2^) for clones K11 and O1.4 across various conditions (basal, TNFα, and TSA) on the *x* axis. The colors representing each clone are indicated in the legend located at the top right of the panel. (**D** and **E**) Bar plot depicting the percentage of extrinsic noise for clones K11 and O1.4 under basal, TNFα, and TSA treatment conditions. The colors corresponding to each condition are provided in the legend at the top right of the panel. (**F**) Schematic representation depicting the paired and unpaired transcription sites.

The extrinsic noise could originate from variations in the extracellular environment or from heterogeneities in the ability of single cells to respond to the inducer. To discriminate between these two possibilities, we designed a procedure to quantify separately these sources of noise (Fig. 3F). We applied the total variance decomposition to two types of reporter copies: those placed in the same cell (“paired” TSs, defining the “total” extrinsic noise; Fig. 3, D to F) and those placed in different cells (“unpaired” TSs, defining the “extracellular” extrinsic noise; Fig. 3, D to F). By subtracting the extracellular extrinsic noise from the total extrinsic noise, one can thus estimate the “intracellular” extrinsic noise, or the part of noise due to cell-to-cell heterogeneity. For both clones, the extracellular extrinsic noise (i.e., from unpaired alleles) did not change between conditions, whereas the total extrinsic noise (i.e., from paired alleles) increased from basal to induced conditions, with a similar effect for TSA and TNFα (Fig. 3, D and E). This suggests that the increase in extrinsic noise following promoter activation was attributable to cell-to-cell heterogeneity. Overall, these data demonstrate that both copies of the integrated MS2 reporter were transcriptionally active and responsive to external stimuli, and that extrinsic noise was readily detectable upon transcriptional stimulation, with a component arising from cellular heterogeneity.

### Live-cell imaging reveals a rapid transient transcriptional stimulation after TNFα, and a slow, continuous increase after TSA treatment

To characterize in detail the transcriptional fluctuations of the HIV-1 promoter, we imaged the two-copy cell lines live. We recorded time-lapse movies for 15 hours at a rate of one three-dimensional (3D) image per minute, using an illumination power enabling the detection of single RNAs throughout the movies (Fig. 4, A and B, and Supplementary Movies). In both basal and after drug treatments, cells exhibited varying and fluctuating levels of transcriptional activity, as illustrated in the snapshots extracted from the movies (Fig. 4, A and B). To further analyze the viral transcriptional behavior, raw live-cell imaging data were processed to extract TS intensities (Fig. 4C). Single RNAs present in the nucleoplasm were used to calibrate the signals such that the exact number of nascent RNAs was calculated at each time point. Examples of trajectories from single cells in the basal condition showed a typical bursting pattern of transcriptional activity, with little apparent temporal correlation between the bursts of the two reporter copies, in either K11 or O1.4 clones (Fig. 4, D and E). Upon activation with TNFα, TSA, or JQ1, transcription activity in single cells remained nonmonotonic and was highly variable between cells. Because of this strong stochasticity, transcription activation was clearly observed in the mean activity of many cells (Fig. 5), but was less apparent in the traces of individual TSs (Fig. 4). Heatmaps displaying the activities of many cells and their averages showed that transcriptional activation raised rapidly after TNFα treatment and culminated after about 30 min with a maximal activation of 16 folds in K11 cells, before returning to basal levels after an additional 30 min (Fig. 5, A and B). In contrast, TSA led to a gradual and sustained increase in transcriptional activity over time, which culminated at about 10 hours for clone O1.4 and 15 hours for clone K11, likely reflecting clonal differences in TSA sensitivity. The maximal fold activation was 19- and 7-fold for clone K11 and O1.4, respectively, in line with the responses observed by smiFISH. We also tested JQ1 and PMA-ionomycin, two other traditional activators of HIV-1. JQ1 is an inhibitor of BET proteins such as BRD4. It blocks their binding to acetylated lysines and has been shown to be a potent activator of latent HIV-1 viruses (*53*). PMA-ionomycin activates protein kinase C and increases calcium levels, which activates viral transcription via NF-kB, NFAT, and AP1 (*54*, *55*). Upon addition of JQ1 in K11 cells, a rapid and sustained increase in transcriptional activity was observed, starting approximately 30 min after addition of the drug, reaching a peak at 95 min and then plateauing for the rest of the movie. In contrast, treatment with PMA-ionomycin resulted in a weak activation with RNA levels remaining close to basal conditions. These observations highlight the diversity of transcriptional responses of HIV1 promoters, shaped by both the nature of the stimulus and, to a lesser extent, clonal variability.

**Fig. 4.**
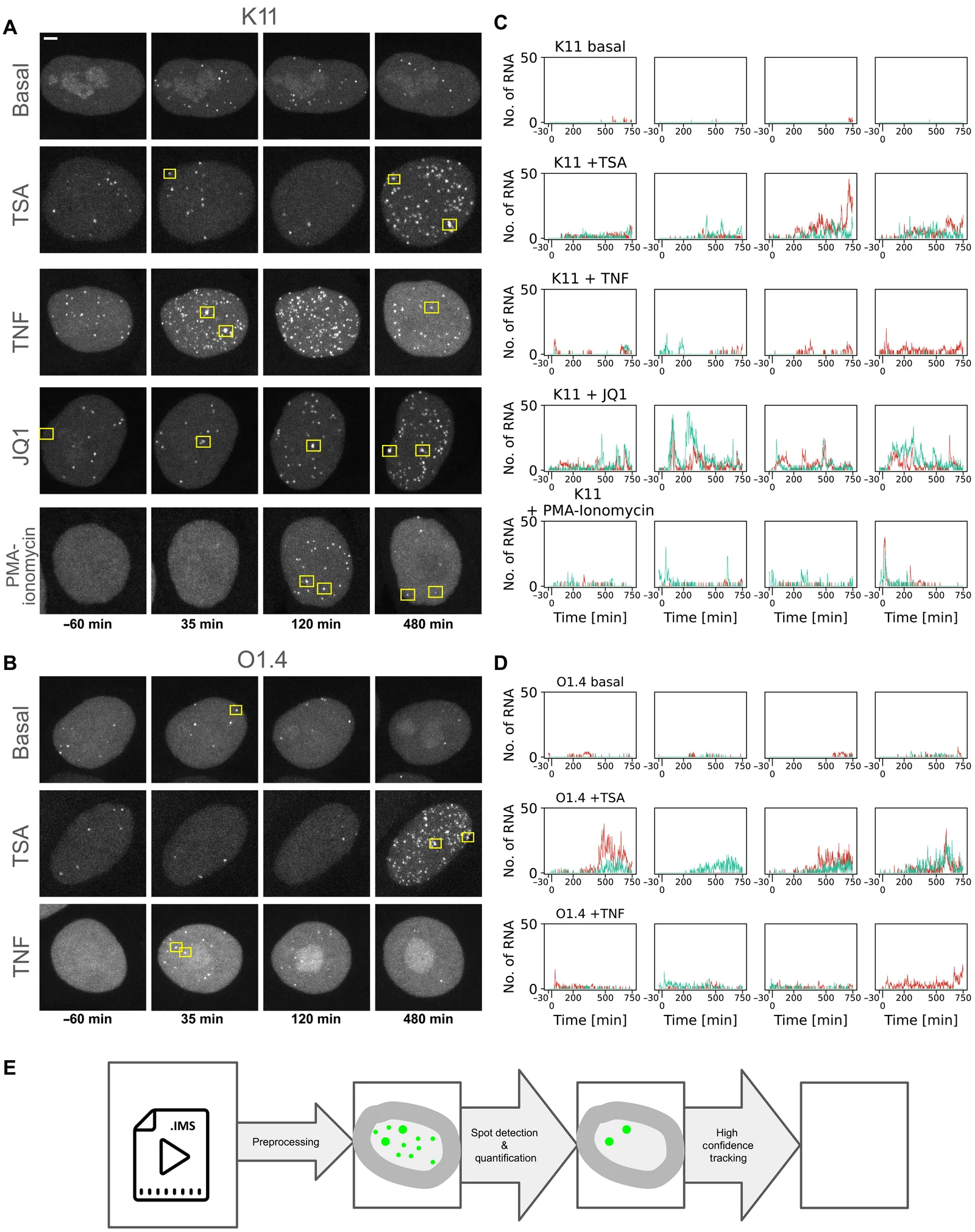
Live-cell analysis of the response of the HIV-1 promoter to diverse activators. (**A** and **B**) Snapshots from live imaging data showing a single nucleus at different time points for clones K11 and O1.4, respectively. From top to bottom, the conditions are Basal, TSA, TNFα, JQ1, and PMA-ionomycin. Yellow squares highlight the activated transcription sites, visible as bright spots with intensities higher than the surrounding spots, where the latter are released single RNAs. Scale bar, 3 μm. (**C** and **D**) Single-cell trajectories of nascent RNA counts per transcription site over time for clones K11 and O1.4, respectively. For each clone, from top to bottom, the conditions are basal, TSA, TNFα, JQ1, and PMA-ionomycin treatment. The *x* axis represents time (in minutes) over a total of 780 min (13 hours), and the *y* axis shows the number of nascent RNA molecules. Each panel is a single cell and its two transcription sites corresponding to the reporter genes are depicted in red and green. (**E**) Workflow illustrating the image analysis method for obtaining transcription site trajectories. The raw data undergoes preprocessing to obtain cropped movies for each nucleus, followed by spot detection, quantification, and high-confidence tracking. The final output is the transcription site RNA count over time.

**Fig. 5.**
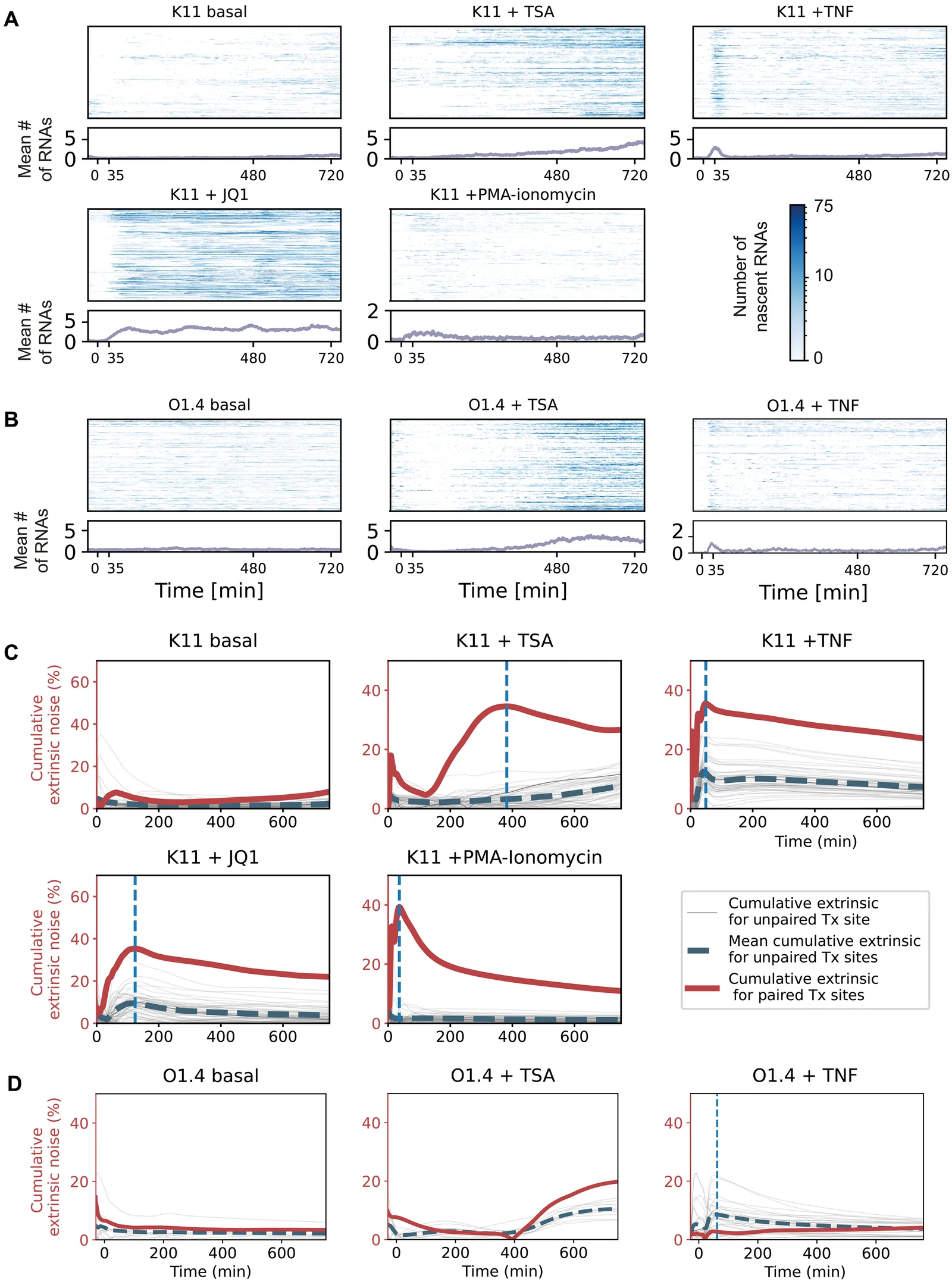
Analysis of drug-induced transcription reveals extrinsic noise and cell heterogeneity. (**A** and **B**) Heatmaps displaying the nascent RNA count over time for clone K11 (A) and clone O1.4 (B), under basal, TSA, TNFα, JQ1, and PMA-ionomycin treatment conditions (as indicated). On the *y* axis, each row represents the RNA count for an individual transcription site, with transcription sites within the same nucleus arranged in adjacent rows. The color bar maps the intensity of the color to the RNA count. Below each heatmap, the mean transcription site intensity is plotted over time. The number of cells analyzed was 39, 107, 76, 164, and 142 for K11 in Basal, TNFα, TSA, PMA, and JQ1, respectively, and 160 and 87 for O1.4 in basal and TSA condition. (**C** and **D**) Cumulative extrinsic noise as a percentage of total noise plotted over time for clones K11 and O1.4 under basal, TSA, TNFα, JQ1, and PMA-ionomycin treatment conditions. The bold red line represents the cumulative extrinsic noise for paired transcription sites as a percentage, while the dashed blue line denotes the averaged cumulative extrinsic noise for unpaired transcription sites. The thin gray lines represent individual cumulative extrinsic noise calculated for a number of randomly selected subpopulations of unpaired transcription sites. The vertical dashed blue line identifies the time where extrinsic noise is maximal in each condition.

### Cumulative analysis of TS activity over time reveals the extrinsic noise in response to promoter activation

TS intensities at a given time provide only a snapshot of promoter activity, as nascent RNAs remain there for only a few minutes (*35*). In the nonstationary regime following application of the treatment, if some sources of noise have short time scales while others operate on long ones, it may be difficult to detect the latter using only TS intensities. To circumvent this issue, we integrated the signal of TSs over time and replaced the instantaneous signal with its corresponding cumulative area under the curve (cAUC). This quantity was used for computing the ‘cumulative’ extrinsic and intrinsic noise by applying the law of total variance to the time-integrated signal (see Materials and Methods, Fig. 5, C and D, and fig. S3). We also computed the “instantaneous” values of noise by calculating noise values using the intensities at each time point (fig. S4, A and B). Both the instantaneous and cumulative extrinsic noise was found to be low in basal condition for the K11 and O1.4 clone. It accounted for less than 5% of the total noise even after integrating the TS signals for 15 hours (Fig. 5, C and D). Thus, the intrinsic fluctuations of promoter activity were almost entirely responsible for expression heterogeneities in the basal state, at short and long timescales. In contrast, we observed a large increase in the cumulative extrinsic noise following TNFα treatment in the K11 clone, peaking at 36% of total noise after 35 min (Fig. 5C). A similar result was obtained with PMA-ionomycin, which also activates viral transcription via signaling pathways. Following the peak, cumulative extrinsic noise then slowly decreased, most likely because the RNAs produced shortly after drug addition provided most of the activity of the TSs even after much longer times. When the cumulative extrinsic noise was computed while starting the cumulative sum at a time point after the TNFα induced peak, it still increased, albeit slowly, reaching 12% after 10 hours (fig. S3). This indicated that besides the ∼1 hour peak of transcription activation, TNFα also has long-term consequences on extrinsic noise, suggesting a memory effect. The initial increase in extrinsic noise seen immediately after TNFα addition was also visible in the instantaneous noise measurements although the data was much noisier (fig. S4A). JQ1 and TSA target chromatin and they led to similar effects as TNFα, but with slower dynamics. JQ1 showed a delayed peak in extrinsic noise at 120 min, with a maximal value of 35% of total noise (Fig. 5C). The delay was much larger for TSA, which led to a late increase in the cumulative extrinsic noise (Fig. 5, C and D), peaking at 35 and 21% of total noise for clones K11 and O1.4, at 6 and 14 hours, respectively. Long-term effects of TSA addition were evident as a steady increase in cumulative extrinsic noise, even when the calculation began 9 hours after TSA was added (fig. S3). These results were confirmed by the instantaneous measures although these data were again much noisier (fig. S4).

As with fixed-cell images, we decomposed extrinsic noise into total and extracellular components by computing the correlation of paired and unpaired TSs, respectively (Fig. 5). In the basal state, the cumulative extrinsic noise was low for both paired and unpaired TSs, and for both the K11 and O1.4 clones (Fig. 5, C and D). However, following stimulation with either any of the activators, and for both the K11 and O1.4 clones, the cumulative extrinsic noise for unpaired TSs (dashed blue plot) was several folds lower than for paired TSs (Fig. 5, C and D), indicating a low extracellular extrinsic component compared to the intracellular extrinsic one (i.e., cell heterogeneity). The strongest differences were seen for K11, with 36 and 3% of total noise being extrinsic for paired and unpaired copies, respectively, at the peak of TSA treatment (400 min), 36% versus 12% after TNFα addition for 35 min (Fig. 5C), 35% versus 9% for JQ1 and 40% versus 3% for PMA, respectively (Fig. 5C). The effect was smaller for the O1.4 clone, but still important, with 25% versus 12% of noise being extrinsic for paired versus unpaired copies at the peak after TSA addition (Fig. 5D). One exception was the O1.4 cells with TNFα treatment, which showed a smaller extrinsic noise for paired alleles than unpaired ones. This was reminiscent of the slight negative correlation measured by smFISH in the same conditions (Fig. 3B), which may be due to a memory effect of promoter state (see Discussion below). In summary, intrinsic noise dominates in the basal state, while drug treatments increase extrinsic noise with distinct dynamics for different signals. The generally higher noise in paired versus unpaired alleles indicates that these effects are mainly due to heterogeneity in the ability of single cells to respond to activators.

The analyses above were conducted on populations of cells. To better evaluate the activities of the two TSs in individual cells under different conditions, we plotted the cumulative intensities (cAUC) of the TSs belonging to the same cell against one another (fig. S5). A linear relationship was observed between the cumulative intensities of the two TSs, which is expected since both signals increase progressively over time. The slope was sometimes away from one. This suggests that the two reporter copies could display differential transcriptional capacity that could be sustained for up to 15 hours.

### Cross-correlation analysis extracts the timescales of extrinsic noise

To better characterize the time-scale of the different noise components, we performed auto-correlation and cross-correlation analyses (see Materials and Methods). We computed the autocorrelation function of the signal of TSs, as well as the cross-correlation function between signals from pairs of sites within the same cell and across different cells. The decomposition laws also apply to these functions, enabling us to distinguish the autocorrelation of intrinsic and extrinsic noise. We further decomposed the extrinsic component into a total, extracellular, and intracellular extrinsic components, using paired and unpaired TSs as above.

In the basal state, a minimal extrinsic component was confirmed for clones K11 and O1.4, as indicated by the alignment of paired and unpaired cross-correlation functions near zero (Fig. 6, A and B, left-most plots). The cross-correlation curves of paired reporters were flat in the basal conditions, indicating that promoter bursting rates were constant, as expected.

**Fig. 6.**
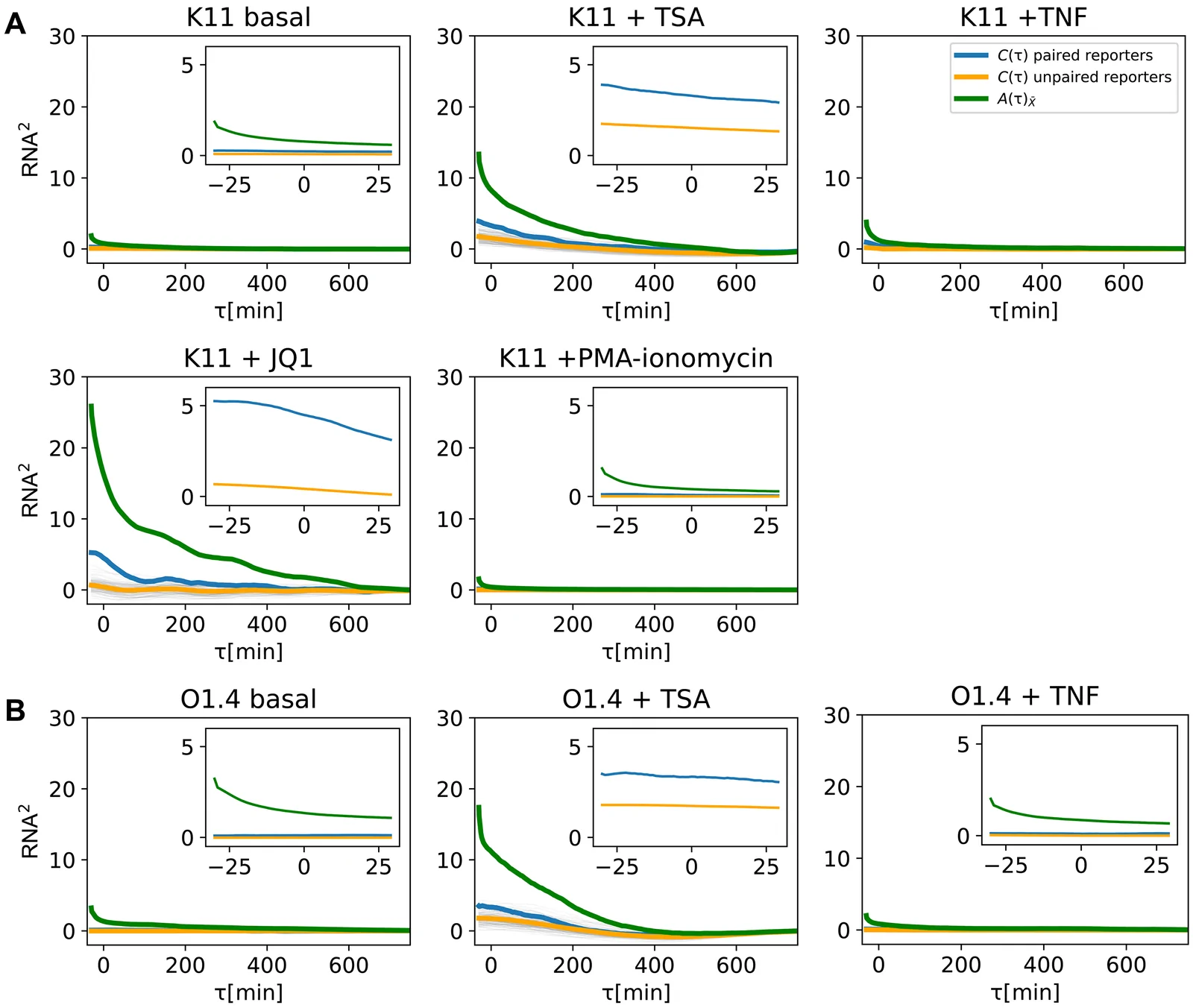
Cross-correlation analyses uncover the timescales of extrinsic and intrinsic noise. (**A** and **B**) Auto- and cross-correlation functions for clones K11 and O1.4 across basal, TSA, TNFα, JQ1, and PMA-ionomycin treatments. The bold green line represents the autocorrelation function of the mean trajectories from two transcription sites within the same nucleus. The bold blue line denotes the cross-correlation of reporter pairs from the same nucleus, while the bold orange line represents the averaged cross-correlation of random reporter pairs sampled over several subpopulations of nuclei. Thin gray lines represent the cross-correlation for each such subpopulation of unpaired sites.

In treated cells, there was a difference between the paired (blue) and unpaired (orange) cross-correlation functions, indicating heterogeneities between cells in both clones (Fig. 6, A and B). This effect was mostly visible for TSA and JQ1, possibly because these were also the strongest inducers (Fig. 5). However, the timescales of cellular heterogeneity varied depending on the signal. To estimate these timescales, we used multiexponential fitting of the difference between paired and unpaired cross-correlation curves (i.e., intracellular extrinsic noise). The fastest response was observed for PMA, with a single exponential fit yielding a timescale of 83 min. The next fastest response was for TNFα treatment, but in this case we found two exponentials corresponding to 93 and 200 min, respectively. JQ1 treatment revealed heterogeneity on a unique long timescale of 195 min. The slowest response was for TSA, corresponding to 230 min in O1.4 and 352 min in K11 clones. This hierarchy of timescales was consistent with what we obtained by the cumulative method in the previous analysis.

In summary, auto- and cross-correlation analyses revealed distinct timescales for extrinsic noise components, with minimal cellular variability in the basal state and drug-specific dynamics of cellular heterogeneity upon stimulation. TSA and JQ1 induced slow, long-term changes in promoter kinetics and long time-scales of cellular heterogeneity, while PMA-ionomycin and TNFα triggered faster, more transient modulations, reflecting their differing regulatory effects on cells and the transcriptional machinery.

### Molecular analysis of drug targets reveals that drug treatment induces cell heterogeneities

Treating the K11 and O1.4 cells with TSA, TNFα, JQ1, and PMA-ionomycin clearly show cell-to-cell differences in viral transcription (Figs. 2 and 5). It also revealed cell heterogeneity, because viral copies in the same cell showed a higher correlation than copies in different cells (Figs. 5C and 6). TNFα acts through a complex signaling cascade activating the canonical NF-kB pathway (*56*–*58*). TSA, instead, modulates chromatin structure by inhibiting histone deacetylases I and II (HDACs) with a resulting increase of acetylated lysines (AcK) and active transcription (*59*, *60*). To gain some insights into the possible origin of the factors conferring heterogeneities in cellular properties, we analyzed several key components of these pathways in single cells, using immunofluorescence (IF) to analyze their localization and fluorescence-activated cell sorting (FACS) to quantify their levels. As shown in Fig. 7A, after 30-min treatment with TNFα, the NK-κB p65 protein translocated into the nuclei as expected. However, the levels of nuclear p65 varied from cell to cell, indicating variable levels and/or timing in the activation of the pathway. Part of this may stem from the TNF receptor itself (TNFR1), as IF and FACS analysis showed that it was expressed to variable levels in cells at the start of the experiment (two-fold difference between the third and first quartile). When analyzing the TSA pathway, lysine acetylation levels increased substantially in the nucleus after TSA treatment, with a regular increase from 2 to 8 hours of treatment (Fig. 7, A and C). The cell-to-cell variability also markedly increased, with a cell-to-cell SD rising from ∼1 to ∼5 over 8 hours. The variability increased even after normalization to the mean, with a peak after 2 hours of TSA treatment (Fig. 7E). The levels of acetylated lysine were also highly uneven with disproportionate representation of high intensity subpopulations, as shown by skewness values ranging from 1.81 to 2.01 and a Gini index from 0.45 to 0.62.

**Fig. 7.**
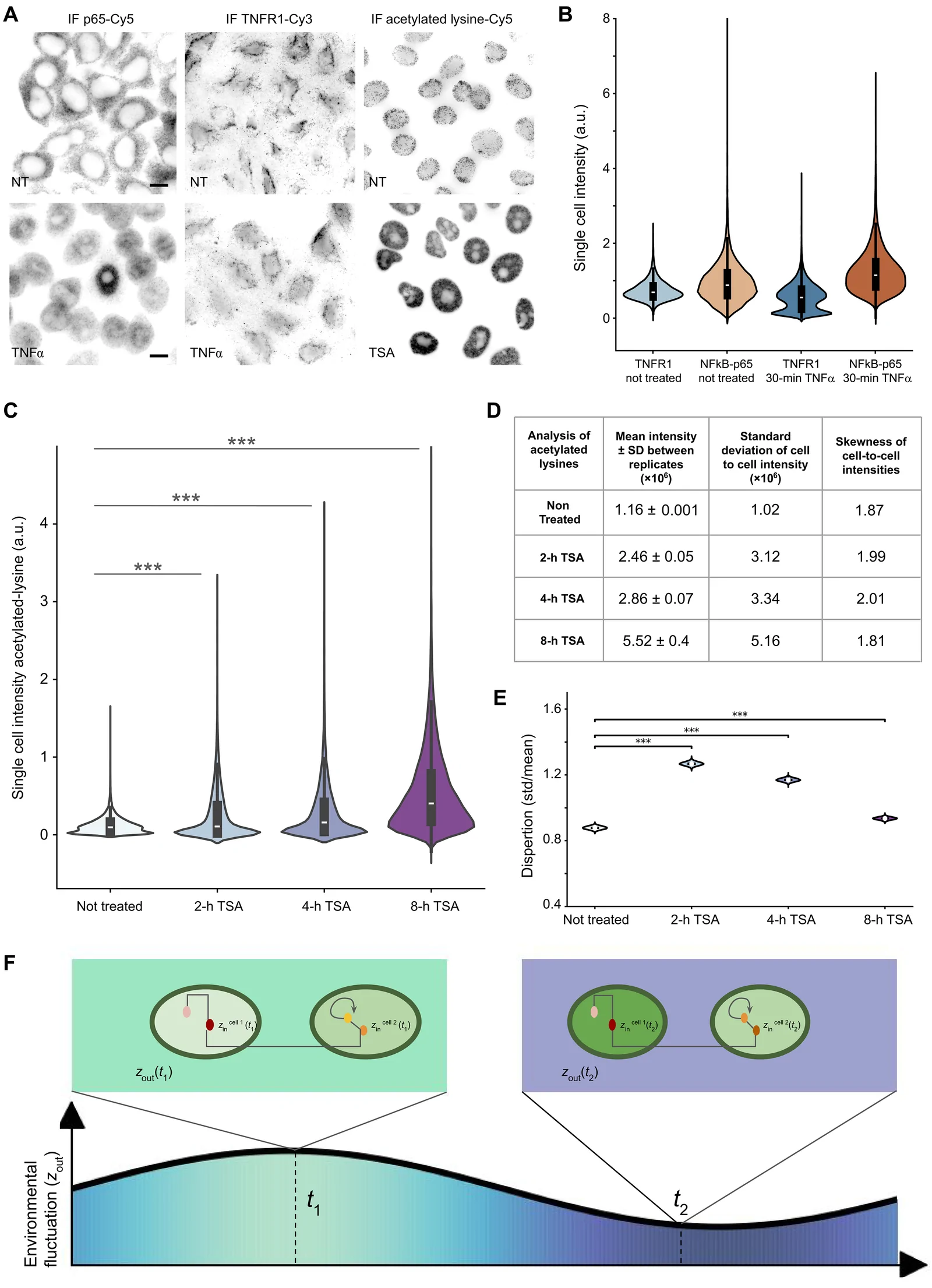
Molecular analysis of cellular heterogeneity. (**A**) IF images of clone K11 under untreated conditions (upper panel) or following treatment with TNFα for 35 min or TSA for 8 hours (lower panel). From left to right, images show IF staining with antibodies against NF-κB p65, TNFR1, and acetylated lysines. Scale bar, 10 μm. (**B**) Violin plot of cytometry data of K11 cells, untreated or treated for 30 min with TNFα and stained with TNFR1 or NFkB-p65 antibodies. The *y* axes represent the cytometry fluorescence intensity measured for each cell (11496 cells for each sample). (**C**) Violin plot of cytometry data of K11 cells, untreated or treated with TSA for the indicated time and stained with an antibody against acetylated lysins. The *y* axis represents the intensity for each cell in two experiments (7913 cells for each experiment; Mann-Whitney *U* test: ***P < 0.001). (**D**). Values of (i) mean intensity ± the SD calculated between replicates (*n* = 2), (ii) SD of cell-to-cell intensities, and (iii) skewness of cell-to-cell intensities. Values are calculated from data in (C). (**E**) Dispersion (SD/mean) of the single cell intensities of acetylated lysins. The *P* values are calculated using the two replicates and a bootstrapping algorithm (7000 samples per group without replacement, ***P < 0.001). (**F**) Sources of extrinsic noise. The shaded plot represents the fluctuation in extracellular extrinsic noise ($Z^{\text{out}}$) shared by several cells, represented by the color of the plot and the color surrounding the cells. Two representative cells, *X* and *Y*, are shown at two distinct time points to depict intracellular extrinsic noise represented by the color inside the cells. The $Z^{\text{in}}$ values reflect the distinct intracellular conditions. Paired transcription sites share the same intracellular and extracellular conditions. Unpaired transcription sites experience the same extracellular parameters but different intracellular conditions.

Together, these data show that cells display important heterogeneities in key components of the NF-κB pathway. More remarkably, TSA treatment appears to generate heterogeneity within the cell population, either de novo or by amplifying pre-existing heterogeneity. These findings highlight that individual cellular properties may contribute substantially to the observed variability in transcriptional activation and are themselves modified by drug treatment.

## DISCUSSION

### Intrinsic promoter dynamics generates expression variability in the basal state

Several studies have used two-copy protein reporters in bacteria, yeast and mammalian cells (*7*, *12*, *61*–*63*). However, the use of protein reporters provides only an indirect measure of transcription, as the long half-lives of the mRNA and their encoded proteins (e.g., 2 hours for destabilized GFPs) tend to buffer transcriptional fluctuations. Real-time imaging of nascent transcripts is currently a low-throughput method requiring extended observation time at high frame rate, often longer than 10 hours with one 3D image per minute. When combined with a two-copy reporter system, it however offers a direct measurement of extrinsic and intrinsic noise sources affecting transcription (*64*). Here, we used cell lines carrying two copies of an HIV-1 reporter tagged with MS2 sites to measure their transcriptional activity in real time, in absence of the viral activator Tat. We found that basal transcription showed a bursting type of expression that was entirely attributable to intrinsic noise: stochastic changes in the molecular states of the promoter that have different abilities to transcribe. This is an important result because, although intrinsic noise is often considered as the main source of noise affecting transcription initiation, only a few studies have directly tested this hypothesis by assessing extrinsic versus intrinsic contributions using two-copy RNA imaging systems (*65*–*67*). These data suggest that for noninducible genes, or inducible genes in the basal states, transcriptional fluctuations are mostly, if not entirely, attributable to the intrinsic dynamic of promoters.

In the case of HIV-1, direct RNA imaging has been used to characterize its stochastic promoter dynamics. The promoter has been shown to fluctuate across multiple time scales, from minutes to hours, a behavior interpreted as intrinsic stochastic switching between a single ON state and several OFF states (*1*, *35*, *36*). An interesting question is how TNFα and TSA modify these intrinsic promoter dynamics to increase transcription rates. TSA inhibits HDACs and the most likely mechanism in this context is chromatin opening, where gene activation corresponds to an increased nucleosome mobility facilitated by TSA. This could be modeled as a promoter with multiple inactive states among which the longest corresponds to closed chromatin. TSA would then destabilize these long inactive periods, resulting in more frequent bursts consistent with our observations (Fig. 5). In the case of TNFα, a previous study using a very similarly tagged HIV-1 system has shown the dependence of burst amplitude on the dose of TNFα (*26*). NF-kB has been shown to interact with multiple coactivators such as CBP and P/CAF (*65*). On HIV-1 specifically, activated NF-kB displaces a repressive HDAC1 complex and recruits P-TEFb to stimulate elongation (*46*, *47*, *66*). By these multiple effects, NF-kB could achieve the initial observed transcriptional pulse and subsequent increase in burst frequency, duration, and amplitude [(*26*) and Fig. 5].

The distribution of RNAs produced by the two copies suggests a slight allelic exclusion effect in the O1.4 clone stimulated with TNFα (Figs. 3B and 5D). As proposed earlier (*68*), this effect could result from stochastic allelic activity. Even for independent copies, if transcriptional bursts are rare, as is the case for O1.4 during TNFα activation, it becomes highly probable that only one copy becomes active during the time window of the observation. This phenomenon can also be interpreted as a memory effect: if promoter dynamics occurs on long timescales, each copy retains its transcriptional state for extended periods. To measure bursting timescales, we used the BurstDECONV method (*36*, *69*) on live MS2 movies of O1.4 cells in the basal state (fig. S6, A and B). BurstDECONV uses the signal of a single RNA polymerase to deconvolve the MS2 time traces and precisely place individual transcription initiation events. Then, the time lag between successive initiation events is used to compute a survival function, which is fitted with a sum of exponentials. The number of exponentials yields the number of promoter states, and their parameters gives the transition rates between these states (*36*, *69*). This analysis confirmed the existence of long inactive timescales, in the order of 1 hour, which is the duration of the activation wave produced by TNFα. Moreover, in a separate work (*70*), we showed using single molecule DNA footprinting that these long bursting timescales of the HIV-1 promoter likely have a chromatin origin.

To confirm a memory effect, we measured how many alleles became active within 1 hour following TNFα addition. In the absence of memory, this number should follow a binomial distribution corresponding to the steady-state behavior of independent Markov chains representing the two alleles. This prediction is well satisfied for the K11 clone stimulated with TNFα, but the state in which only one allele is active is overrepresented in O1.4 clones stimulated with TNFα, confirming the presence of memory effects in the latter case (fig. S6, C and D). Such a memory effect is reminiscent of the case of multiple sequential TNFα stimulations, where a memory effect explains why the HIV-1 promoter becomes more susceptible to TNFα treatment after it responds positively to a first TNFα application (*26*).

### Cellular heterogeneity accounts for variable responses to LRAs

In contrast to the basal state, extrinsic noise became readily detectable when the promoter was activated with TNFα or LRAs such as TSA, JQ1 or PMA-ionomycin. It accounted for up to 40% of total noise and displayed differences depending on the inducer or the cell clone. While RNA imaging has been extensively used to study intrinsic noise in yeast, *Drosophila*, and human cells, studies investigating extrinsic noise sources remain scarce. Only two previous studies have used live-cell imaging of RNA in combination with a two-copy system to infer intrinsic and extrinsic noise contributions (*65*–*67*). Both studies used estrogen responsive genes, TFF1 and GREB1, and homozygous endogenous tagging with MS2 or PP7 sites to generate a homozygous tagged cell line that was imaged in presence of a subsaturating amount of estrogen. Extrinsic noise was detected and accounted for the majority (*66*) or a minority of the total noise (*67*). Combined with our study, these results collectively suggest that, while intrinsic noise dominates expression variability in basal states, extrinsic noise becomes more apparent during transcriptional stimulation and is then responsible for a large portion of expression heterogeneity.

While considering sources of extrinsic noise, it is conceptually useful to distinguish between distinct categories. Here, we separate intracellular extrinsic noise, which encompasses factors that are specific to individual cells such as variation in transcription factor levels, metabolic state, or cell cycle stage, from extracellular extrinsic noise, which refers to shared fluctuation that uniformly affects all cells in a population, such as changes in temperature, nutrient availability or extracellular signaling cues. This distinction has important implications for interpreting transcriptional heterogeneity and can be assessed by comparing gene copies in the same cell versus different cells. In the case of HIV-1, our results indicate that heterogeneity in the ability of single cells to respond to the activators play a major role in the variability of the viral transcriptional responses. Differences in the properties of individual cells were observed with all the compounds we tested (TNFα, TSA, JQ1, and PMA-ionomycin), which include both natural activators and synthetic drugs, suggesting that this may be a broadly general phenomenon. This raises a fundamental issue in the case of HIV-1 because cells failing to respond to activators remain in the body and provide the reservoir responsible for future viral rebounds. While ample evidence has shown that the intrinsic dynamics of the viral promoter can affect latency entry and exit (*4*, *18*, *20*), our results indicate that the heterogeneity of individual cells to sense and respond to viral activators also contribute to reservoir dynamics in patients, and that it contributes to the viral heterogeneous response to LRAs, which limits the efficacy of curing strategies such as shock-and-kill. Nailing down the causes of the heterogenous properties of single cells is thus an important issue for therapeutic approaches targeting the viral reservoir.

### Possible origins of cellular heterogeneity

The different sensitivity of single cells to viral activators may be fundamental for HIV-1 latency control and this raises the question of its origin. In the case of TNFα, it has been shown that signaling induces oscillations in NF-kB nuclear localization and that the heterogeneity of the response to TNFα is dependent on its dosage and stimulus duration (*71*). It has also been proposed that the response heterogeneity may arise from a variable abundance of TNFα receptors at the cell surface (*72*), a feature apparent in our cell system, as well as from negative and feed forward loops in the pathway that are designed to enhance heterogeneity (*73*). It has been suggested that the TNFα pathway has built-in characteristics that generate heterogeneities even in isogenic populations, which result from an evolutionary trade-off between the conflicting needs to respond efficiently to infection while avoiding too much cell death (*72*). A heterogeneous response allows the pathway to rapidly kill infected cells while protecting many noninfected ones. Our results additionally indicate that noise due to the intrinsic dynamic of the target promoters also contributes to the heterogeneity of the response to TNFα.

TSA inhibits HDACs, a family of four classes of enzymes comprising 18 members that catalyze the removal of acetyl groups from lysines, in particular on histone tails. TSA leads to chromatin decondensation and transcriptional activation, including for HIV-1 (*74*). The variability of the viral response to TSA showed a strong contribution of intracellular extrinsic noise, indicating different abilities of individual cells to respond to the drug. This was observed in both the K11 and O1.4 clones, although with a stronger effect and earlier kinetics for K11. These differences in kinetics are likely due to clonal variability, which indirectly also underscores the heterogeneity in the properties of individual cells in the parental population. Given the role of noise for HIV-1 biology (*4*, *20*, *22*), it would be important to determine the exact cellular properties that make cells sense and respond differently to LRAs molecules like TSA. Measurement of acetylated lysines in cells, the direct molecular consequence of HDAC inhibition, shows that TSA greatly increases the cell-to-cell variability in acetylated lysines, a visible and possibly long-lasting manifestation of the variable properties of individual cells detected by the two-copy imaging system. It is unclear how TSA could create this heterogeneity. The histone deacetylase HDAC1, a direct target of TSA, has been recently shown to be rapidly and strongly up-regulated in response to TSA (*75*), suggesting a negative feedback loop in which cells respond to globally limit the effect of the inhibitor. Possibly, intrinsic noise in HDAC1 transcriptional response to TSA may translate into variable HDAC1 levels between cells, which becomes a global property of the cell that produces extrinsic noise for HDAC1 target genes. Similarly, it has been shown that TSA up-regulates multidrug resistance genes (*76*, *77*), which may similarly convert an intrinsic noise into an extrinsic one, i.e., variable intracellular TSA concentrations. This would also yield heterogeneous cellular properties that can shape their viral response to the drug. In the future, it will be interesting to test these hypotheses, for TSA and other drugs used as LRAs, with the aim of improving their therapeutic efficacy.

The existence of transcriptionally inactive latent subpopulations poses a major barrier to the efficacy of shock-and-kill HIV-1 cure strategies. Enhancing the uniformity of cellular responses and increasing the proportion of activated cells could, therefore, improve therapeutic outcomes. While the intrinsic component of heterogeneity, driven by stochastic promoter switches, remains challenging to modulate, our results demonstrate that a substantial fraction of heterogeneity (up to 40%) stems from cell heterogeneity at the levels of signal transduction and phenotypic penetrance of drugs. These extrinsic components may be governed by molecular factors whose abundance and activity may be, at least in principle, amenable to modulation. Identifying these regulatory determinants is thus of critical importance to improve therapeutic strategies targeting the viral reservoir.

## MATERIALS AND METHODS

### Cell culture and construction of CRISPR cassettes

HeLa H9 cells (gift of S. Emiliani, Cohin Institute, Paris) were grown in Dulbecco’s modified Eagle’s medium (DMEM; Gibco, no. 31966021) supplemented with 10% fetal bovine serum (Sigma-Aldrich) and penicillin/streptomycin (1/100 U/ml; Gibco, no. 15140-122).

The HIV-1 reporter constructs were generated from the pIntro plasmid, which contains an optimized HIV-1 reporter with 128 MS2 loops (*35*). The pIntro plasmid also includes a 1500 bp stretch of human genomic DNA corresponding to a real HIV-1 integration site from a patient, located within the TBC1D5 gene and which corresponds to the right homology arm. In addition, we cloned a 500 bp left homology arm upstream of the HIV-1 reporter. Downstream of this second homology arm, we incorporated a polylinker to clone two different selection markers, one for each repair cassette. In one cassette, we used a blasticidin selection marker under the control of the EF1α constitutive promoter. In the second cassette, we replaced the blasticidin marker with the hygromycin marker from the ptv-oct4 plasmid (Addgene, no. 62351). Both selection markers were inserted in the reverse orientation relative to the HIV-1 LTR promoter. Plasmid sequences are available on request.

### Generation of HeLa CRISPR MCP-tdStayGold clones

To insert the HIV-1 reporters into HeLa cells, we used one Cas9 guide RNA directing cleavage in between the two homology arms of the HIV-1 repair plasmids. The PAM and the guide binding sites were absent from the repair cassettes. The sequence of the sgRNA was 5′ TGGGTTACTCCTGAGTTAAC and it was cloned in pUC57-attbU6 sgRNA vector (*78*). Cas9-nickase expressing plasmid was provided from (Addgene, no. 42335). Plasmids were transfected in a 3:3:1 ratio (repair cassette, U6 sgRNA, Cas9 Nickase) using Jet Prime (Polyplus, no. 114-75), and cells were split after 24 hours and put under selection. Two weeks after transfection, individual clones were picked using cloning discs (Merck, no. F37847-0001) and cultured in 24-well plates under Hygromycin selection at 150 μg/ml final concentration following the first round of CRISPR transfection, and additionally under blasticidin selection at 8 μg/ml following the second round of transfection. Clones were verified by genotyping and smiFISH after each round of transfection. Following the second CRISPR transfection, single-cell clones were sorted to ensure clonal populations, the selected clones were again genotyped and verified by smiFISH. Selected HeLa clones were transduced with a lentivirus expressing NLS-MCP-tdStayGold (*48*) and FACS sorted to ensure stable expression.

### SmiFISH, IF, and fixed-cell image acquisition

SmiFISH was performed as described previously (*79*). In brief, 2.5 × 10^5^ cells were seeded on 22 × 22 mm glass coverslips. At 70% confluency, cells were fixed with 4% paraformaldehyde (PFA) in PBS for 20 min and permeabilized overnight with 70% ethanol at 4°C. Permeabilized cells were washed twice with PBS and incubated for 30 min at room temperature in a solution containing 15% formamide in 1× SSC. Unlabeled primary oligonucleotides were prehybridized with Cy3- or Cy5-labeled secondary oligonucleotides in a solution containing 100 mM NaCl, 50 mM tris-HCl, and 10 mM MgCl_2_ (pH 7.9), for 3 min at 65°C and 10 min at room temperature to form a labeled duplex. Cells were hybridized overnight at 37°C with the duplex in the following mix: 15% formamide, tRNA (0.34 mg/μl), RNase-free bovine serum albumin (BSA) (0.2 mg/ml), 2 mM VRC, 1× SSC, and 10% dextran sulphate. Cells were washed three times for 45 min in 15% formamide 1× SSC, washed three times 1 min with PBS, and the slides were mounted in Vectashield containing 4′,6-diamidino-2-phenylindole (DAPI; Vector Laboratories). Probe sequences are given in spreadsheet S3.

For IF, cells were incubated in 0.2% Triton X-100 in PBS for 20 min. Cells were first blocked with 3% BSA in PBS for 30 min at room temperature. Following blocking, cells were incubated for 1 hour 30 min at room temperature with primary antibodies diluted in PBS containing 0.1% BSA; anti–NF-κB p65 (Cell Signaling Technology, no. D14E12) at a 1:800 dilution, anti-TNFR1 (Proteintech, no. 60192-1-Ig) at a 1:100 dilution, and anti-acetylated lysine (Thermo Fisher Scientific, no. MA1-2021) at a 1:500 dilution.

Cells were then washed 3× with 1× PBS, 10 min each. Following washes, cells were incubated with fluorescently labeled secondary antibodies: Cy5-conjugated goat anti-rabbit IgG (Jackson ImmunoResearch, no. 111-175-144; 1:200) for NF-κB p65, Cy5-conjugated donkey anti-mouse IgG (Jackson ImmunoResearch, no. 715-166-150; 1:200) for acetylated lysine, and Cy3-conjugated donkey anti-mouse IgG (Jackson ImmunoResearch, no. 711-166-152; 1:200) for TNFR1.

Images were acquired using an upright fluorescent wide-field microscope (Zeiss Axioimager Z2 apotome). Images were taken using 63× objective [Apochromat 1.46 numerical aperture (NA) oil Corr], using Zen 3.10 software. Multidimensional acquisition was used to acquire 3D Z-stacks in 4 channels: DAPI, GFP, Cy3, and Cy5. The distance between each image in the stack was set to 0.3 μm. Maximum projection was performed to transform Z-stacks into 2D images. Figures were prepared with ImageJ, OMERO, Adobe Photoshop, and Illustrator.

### Flow cytometry measurements and analysis

Hela K11 cells were plated in 10-cm dishes the day before the experiment. The next day cells were treated with TNFα (10 ng/ml) or TSA (400 nM) for the indicated time, fixed in 4% PFA and permeabilized with 0.2% Tween in 1× PBS (except for TNFR1 labeling). The cells were incubated overnight at 4°C either with NF-kB p65 polyclonal antibody (1:100, Thermo Fisher Scientific, no. 51-0500), TNF receptor 1 monoclonal antibody (TNFR1; 0.2 μg per 10^6^ cells; Proteintech, no. 60192-1-Ig) or acetylated lysine monoclonal antibody (1:200, Thermo Fisher Scientific, no. MA1-2021). After washing, the cells were stained with the appropriate Cy5 secondary antibody (1:200; goat anti-rabbit IgG, Jackson ImmunoResearch, no. 111-175-144 or goat anti-mouse IgG, Jackson ImmunoResearch, no. 115-175-166) and DAPI (5 μg/ml) for 1 hour at room temperature. The cells were analyzed with the NovoCyte Quanteon Flow Cytometer Systems 4 Lasers (Agilent) setting the filters R667 (ex/em, 637/667 ± 30) for Cy5 and V445 (ex/em, 405/445 ± 45) for DAPI. Quantifications were done using FloJo v10.10.0. The intensity values were first assessed for normality and unimodality. A Mann-Whitney *U* test was performed between replicates to evaluate their similarity; upon confirmation of no significant differences, the replicates were pooled. From this aggregated dataset of 15826 cells, a subpopulation of approximately 7000 values was randomly sampled to calculate the SD and a dispersion metric (SD/mean). This resampling procedure was repeated 10,000 times to generate a distribution of dispersion values. A two-sample *t* test was subsequently conducted to compare experimental groups and assess significant deviations from the null hypothesis.

### Live-cell image acquisition

Cells were plated on 35-mm glass-bottom imaging dishes “Fluorodish” (WPI, no. FD35-100) and grown at 37°C to reach 70% confluence (usually within 48 to 72 hours). Before image acquisition, the cells were washed twice with PBS, and the culture medium was replaced with live-cell imaging medium (Fluorobrite DMEM; Thermo Fisher Scientific, no. A1896701). The medium was supplemented with 1× GlutaMAX, 10% FBS, 500 μM ascorbic acid (Sigma-Aldrich, no. A92902), 3 μM rutin (Evrogen, no. MCK02), and live-cell antifade reagent (Thermo Fisher Scientific, no. P36974). Cells were then equilibrated in the microscopy incubation chamber for at least 20 min at 37°C and 5% CO_2_ before imaging.

Movie acquisition was conducted using an inverted Nikon microscope coupled to a Dragonfly spinning disk (Andor), equipped with a 100× Plan Apo 1.45 NA oil immersion objective. Excitation was achieved using a 488-nm laser, with an exposure time of 75 ms at 5% laser power in the GFP channel. Images were captured on an EMCCD iXon888 camera (1024 × 1024 pixels, effective pixel size: 0.121 μm) in multiposition acquisition mode. Z-stacks consisting of 13 to 15 optical slices, each separated by 0.6 μm, were acquired every 1 min for a total of 13.5 hours. The perfect focus system was used, and 10 to 12 different stage positions were imaged during each session. No signs of cellular stress observed during the imaging period and cells divided normally while imaged.

For drug treatments, TNFα, TSA, JQ1, and PMA-ionomycin were introduced to the plate on-stage through a small opening in the plate cover, after 1 hour of imaging in basal conditions. The drug of interest, dissolved in an equal volume of Fluorobrite, was added dropwise to the medium. The concentrations of the drugs were as follows: TNFα (10 ng/ml), TSA (400 nM), JQ1 (1 μM), and PMA/ionomycin (100 nM/2 μM).

### RNA quantification from smiFISH image data

SmiFISH analysis was performed using a friendly user application called “SmallFISH,” which is accessible through Zenodo: https://doi.org/10.5281/zenodo.20562793. It operates by integrating cell segmentation using Cellpose, a deep learning–based algorithm (*80*), with single-molecule detection through BigFISH, which uses a noise-robust local maxima filter for precise detection (*81*). The software then extracts a variety of features for each cell (number of RNA and TS intensities), enabling statistical analysis across large batches of cells. To detect TSs, an intensity threshold equivalent to the brightness of two RNAs was applied. Raw RNA counts per cell are given in in spreadsheet S1.

### Live-cell quantifications

Quantification of TSs intensities from raw microscopy time-lapse imaging data was performed in several steps.

#### Preprocessing

We performed maximum intensity projections along both the *Z* axis and the temporal axis for each field of view to outline the overall spatial footprint of each nucleus. Nuclear segmentation was subsequently carried out at each time point using an automated pipeline based on Cellpose (*80*). This pipeline also enabled cropping of individual nuclei and temporal alignment of their centroids to generate motion-corrected time-lapse image series for all nuclei within a field of view. Photobleaching was quantified by measuring the sum of pixel intensities within nuclear masks and comparing these values to intensities measured in background regions. For three-dimensional image stacks exhibiting greater than 20% photobleaching, correction was performed using the bleach correction plugin in Napari. Missing frames were imputed by calculating the mean image from adjacent time points (*t* − 1 and *t* + 1).

#### Spot detection and TS quantification

Spot detection thresholds and clustering parameters were empirically optimized for each cell from a sample of frames spaced across the temporal dimension. Using the Spot and Cluster Detection module of liveQuant (https://doi.org/10.5281/zenodo.20562702), spots were detected across all frames. The detected spots were then used to construct a reference spot representing the fluorescence signal of a single RNA molecule. This reference model was then used to decompose detected clusters into estimated counts of individual RNA molecules, as done in bigFISH (*81*). TS positions were initially detected in each frame by applying a computational blurring technique designed to attenuate single-molecule signals while preserving the transcription site signal. These generated tracks that were then manually corrected and used to obtain high confidence transcription site signals. Raw counts of nascent RNA per transcription site are given in in spreadsheet S2.

### Data preparation and noise analysis

#### Total variance decomposition to obtain total, extracellular, and intracellular extrinsic noise components

Consider a two-reporter system with two transcription sites like in Fig. 7F. The two sites function independently but both collect signals from the environment. We suppose that the sites are otherwise equivalent and contain replicas of the same promoter and cis-regulatory regions. In order to quantify extrinsic noise, we consider the covariance of the RNAs produced by two different sites. We consider two situations: (i) paired sites, when the two sites are chosen in the same cell and (ii) unpaired sites, when the two sites are chosen in different cells. By doing so we probe two types of extrinsic noise: intracellular and extracellular. Intracellular sources affect each cell differently, while extracellular sources affect groups of cells identically. These latter sources include changes in the external environment, such as signaling molecules and drugs. Intracellular sources involve heterogeneity in transcription factors levels, metabolism, responses to signaling molecules, and cell-cycle stages.

Adapting reasonings from (*9*, *82*, *83*) we formalize the problem as follows:

1) The variable $Z^{\text{in}}$ represents the cellular internal environment and the variable $Z^{\text{out}}$ represents the external environment shared by several cells. Let x be the measured gene product (nascent, or total RNA).

2) The extrinsic noise is represented by the conditional expectation ${\bar{x}}_{\text{extr}}=E\left[x|Z^{\text{in}},Z^{\text{out}}\right]$, i.e., the mean expression at fixed internal and external environment

3) The extracellular extrinsic noise is ${\bar{x}}_{\text{extr},\text{out}}=E\left[x|Z^{\text{out}}\right]$, i.e., the mean expression at a fixed external environment.

4) The intracellular extrinsic noise is ${\bar{x}}_{\text{extr},\text{in}}={\bar{x}}_{\text{extr}}-{\bar{x}}_{\text{extr},\text{out}}$.

5) Last, the intrinsic noise is ${\bar{x}}_{\text{intr}}=x-{\bar{x}}_{\text{extr}}$.

The tower property of the conditional expectation, also known as the law of total variance leads to

$$\text{Var}\left(x\right)=\text{Var}({\bar{x}}_{\text{extr}})+E\left[\text{Var}\left({\bar{x}}_{\text{intr}}\right)\right]$$ (1)

Furthermore,

$$\text{Var}\left({\bar{x}}_{\text{extr}}\right)=\text{Var}\left({\bar{x}}_{\text{extr},\text{out}}\right)+E\left[\text{Var}\left({\bar{x}}_{\text{extr},\text{in}}\right)\right]$$ (2)

where the expectation is over all values of the external environment.

Adapting results from (*9*, *82*, *83*) to our experimental design, the covariance of unpaired sites can be used to estimate $\text{Var}\left({\bar{x}}_{\text{extr},\text{out}}\right)$, whereas the covariance of paired sites provides $\text{Var}({\bar{x}}_{\text{extr}})$.

According to (1) and (2), the variance of single site signal decomposes in three components: intrinsic, intracellular extrinsic and extracellular extrinsic noise.

#### Fixed-cell experiments

The mean counts of nascent RNA per transcription site and released RNA per nucleus for fixed-cell data were calculated and presented as histograms, which were plotted using the Seaborn Python data visualization package. In addition, correlation plots between the two transcription sites for each condition were generated for each clone, providing further insight into the relationship between the transcription dynamics. The extrinsic and intrinsic noises were computed using the law of total variance (see Eqs. 1 and 2). Using (*9*, *82*, *83*), the contributions of extrinsic and intrinsic noise to total noise were estimated using the following formulas

$$\eta_{\text{intrinsic}}^{2}=\frac{\frac{1}{n}\left[\sum_{i=1}^{n}\frac{1}{2}{\left(x_{i}-y_{i}\right)}^{2}\right]}{\bar{x}.\bar{y}}$$ (3)

$$\eta_{\text{extrinsic}}^{2}=\frac{\frac{1}{n}\sum_{i=1}^{n}x_{i}.y_{i}-\bar{x}.\bar{y}}{\bar{x}.\bar{y}}$$ (4)

where $x_{i},y_{i}$ are values of mRNA numbers for pairs of transcription sites, and $\bar{x},\bar{y}$ are the respective means. In Eq. 3 the sum is over both true pairs (same cell transcription sites) and simulated random pairing (transcription sites from different cells). When in Eq. 4 the sum is over true pairs, $\eta_{\text{extrinsic}}^{2}$ estimates the intracellular and extracellular extrinsic noise, whereas a sum over unpaired sites in Eq. 4 estimates only the extracellular extrinsic noise, where *n* is the number of pairs.

#### Live-cell experiments

Each dataset consists of time-resolved traces of TS RNA copy number, from multiple cells under distinct experimental conditions, such as basal or drug-treated. A custom Python script was used to compute cumulative noise metrics using a time cumulative approach that incorporates data from preceding time points. This method enables the quantification of intrinsic, extrinsic, and total noise components as they evolve over time. Formulas 3 and 4 were thus modified to account for the temporal evolution of the noise as follows

$$\eta_{\text{intrinsic}}^{2}\left(t^{\prime }\right)=\frac{\frac{1}{M_{t'}}\sum_{t=1}^{t\leq t^{\prime }}\sum_{i=1}^{n}\frac{1}{2}{\left(x_{i}^{t}-y_{i}^{t}\right)}^{2}}{\left[\frac{1}{M_{t'}}\sum_{t=1}^{t\leq t^{\prime }}\sum_{i=1}^{n}x_{i}^{t}\right]\left[\frac{1}{M_{t'}}\sum_{t=1}^{t\leq t^{\prime }}\sum_{i=1}^{n}y_{i}^{t}\right]}$$ (5)

$$\eta_{\text{extrinsic}}^{2}\left(t^{\prime }\right)=\frac{\frac{1}{M_{t'}}\sum_{t=1}^{t\leq t^{\prime }}\sum_{i=1}^{n}x_{i}^{t}.y_{i}^{t}-\left[\frac{1}{M_{t'}}\sum_{t=1}^{t\leq t^{\prime }}\sum_{i=1}^{n}x_{i}^{t}\right]\left[\frac{1}{M_{t'}}\sum_{t=1}^{t\leq t^{\prime }}\sum_{i=1}^{n}y_{i}^{t}\right]}{\left[\frac{1}{M_{t'}}\sum_{t=1}^{t\leq t^{\prime }}\sum_{i=1}^{n}x_{i}^{t}\right]\left[\frac{1}{M_{t'}}\sum_{t=1}^{t\leq t^{\prime }}\sum_{i=1}^{n}y_{i}^{t}\right]}$$ (6)

$$\eta_{\text{Total}}^{2}\left(t^{\prime }\right)=\eta_{\text{extrinsic}}^{2}\left(t^{\prime }\right)+\eta_{\text{intrinsic}}^{2}\left(t^{\prime }\right)$$ (7)

Here, $x_{i}^{t}$, $y_{i}^{t}$ represent the mRNA values from the two transcription sites at a particular time point *t*, and *M_t′_* is the number of data points with $t\leq t^{\prime }$. This cumulative approach enables a progressively refined assessment of noise contributions, capturing temporal trends in transcriptional variability and avoiding the limitations of analyzing isolated time points, as instantaneous estimators are less robust and exhibit greater variability. To demonstrate this, instantaneous noise metrics were also calculated by considering data from individual time points. A moving average (window size = 5) of these instantaneous noise values was applied to reduce the impact of outliers, along with an overlay of the cumulative values of true pairs. The code for computing the instantaneous and cumulative noise is available on Zenodo: https://doi.org/10.5281/zenodo.20562731.

For the autocorrelation analysis, the autocorrelation function of each trace and the cross-correlation functions between two true paired (same cell TS) or unpaired traces (TS from different cells) were defined as

$$R_{\mathrm{xx}}\left(\tau \right)=\frac{1}{N-\tau }\sum_{t=0}^{N-\tau }x\left(t\right)x\left(t+\tau \right)$$ (8)

and were calculated using the inverse fast Fourier transform F as

$$R_{\mathrm{xx}}\left(\tau \right)=F^{-1}\left\{{|F\left\{x\left(t\right)\right\}|}^{2}\right\}$$ (9)

The average correlation functions were then plotted for each condition and clone. These analyses were performed using custom Python scripts, using the SciPy and NumPy libraries. To eliminate bias in selecting the “first site,” the RNA trajectories were shuffled. In addition, for computing the correlation function of unpaired traces, transcription sites were shuffled such that two randomly selected transcription sites were never from the same nucleus. A bootstrap of 50 random permutations of pairs of transcription site trajectories equal to the total number of cells were performed to obtain an average cross-correlation function for unpaired traces (Fig. 6, A and B).

The autocorrelation functions contain information about the timescales of the noise, which can be extracted through multiexponential regression. Specifically, we use the following parametric model

$$R_{\mathrm{xx}}\left(\tau \right)=\sum_{i=1}^{m}A_{i}e^{-\tau /t_{i}}$$ (10)

where $t_{i}>0$ are timescales and $A_{i}$ are other parameters.

The number of exponentials is determined by a parsimony criterion. Starting with *m* = 1, we progressively increase *m* until the least squares error of the regression becomes sufficiently small, while ensuring that the uncertainty in the timescale parameters remains limited, in order to avoid overfitting. The uncertainty error is estimated by computing a set of optimal and suboptimal solutions to the regression problem, and taking the minimum and maximum parameter values within this set.

In Eq. 9, we assume that the exponents are real. However, cases involving complex exponents and oscillatory behavior of the autocorrelation function are also encompassed by the same formula, when *A_i_* and *t_i_* are complex parameters. Since the number of real parameters doubles in such cases (each complex parameter has real and imaginary parts), we did not consider them to avoid overfitting.

## Supplementary Materials

### The PDF file includes:

Figs. S1 to S6 Legends for spreadsheets S1 to S3 Legends for movies S1 to S7

### Other Supplementary Material for this manuscript includes the following:

Spreadsheets S1 to S3
