# Supplementary material for: Cell heterogeneity contributes to the variable response of HIV-1 to latency-reversing agents
Source: Sci Adv. 2026 Oct 9;12(41):eaeb1435. doi: 10.1126/sciadv.aeb1435 (PMC13651875; doi:10.1126/sciadv.aeb1435)
Supplement: Supplementary file 1 [file sciadv.aeb1435_sm.pdf]

Science Advances
AAAS

Supplementary Materials for

Cell heterogeneity contributes to the variable response of HIV-1 to latency-reversing agents

Rachel Topno *et al.*

Corresponding author: Ovidiu Radulescu, ovidiu.radulescu@umontpellier.fr;
Edouard Bertrand, edouard.bertrand@igh.cnrs.fr

Sci. Adv. **12**, eaeb1435 (2026)
DOI: 10.1126/sciadv.aeb1435

The PDF file includes:

Figs. S1 to S6
Legends for spreadsheets S1 to S3
Legends for movies S1 to S7

Other Supplementary Material for this manuscript includes the following:

Spreadsheets S1 to S3
Movies S1 to S7

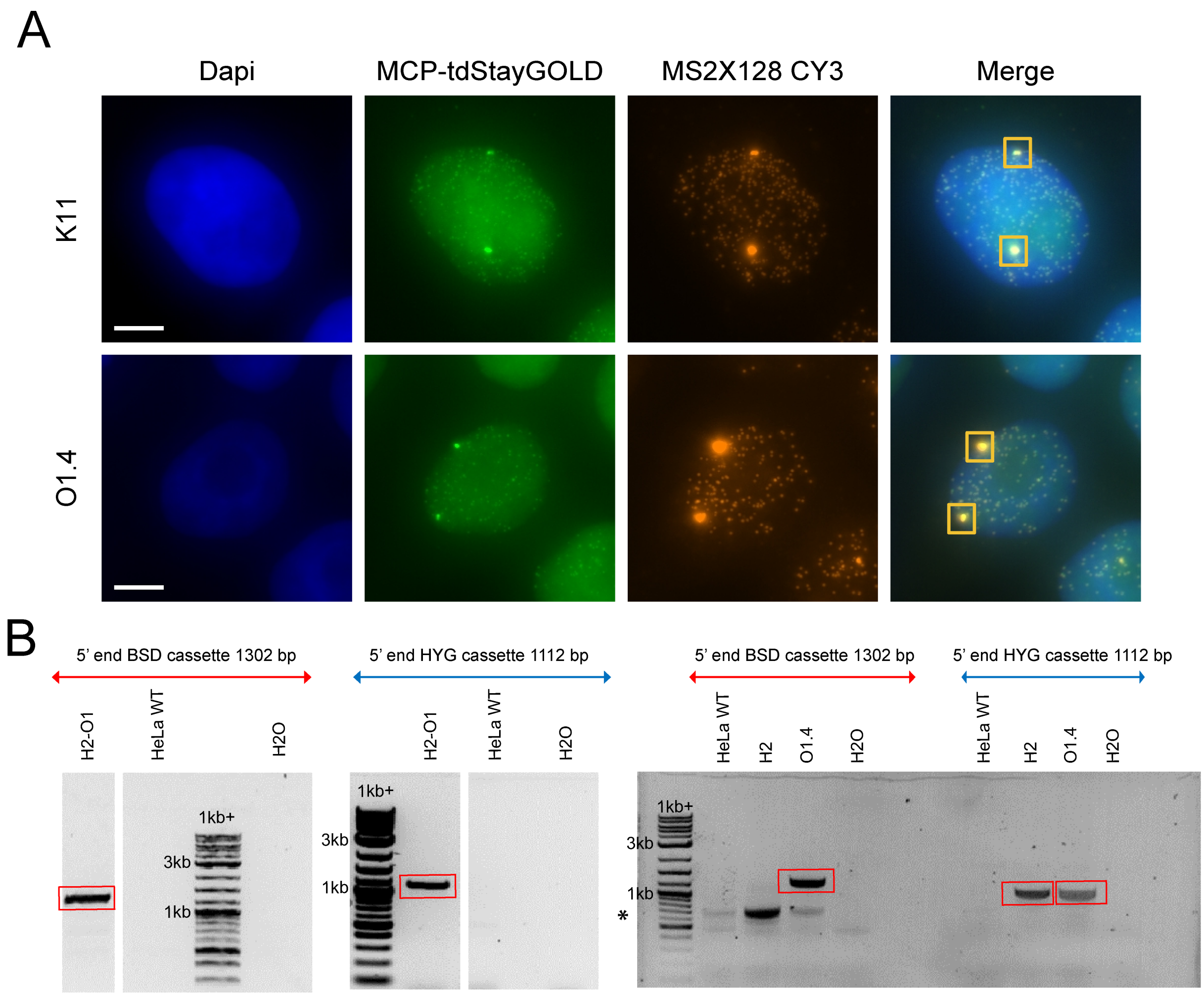

Supplementary Figure 1. Characterization of the two-copy HIV-1 CRISPR clones.

A. Micrographs of K11 (upper panel) and O1.4 (lower panel) cells, 8 hours after treatment with TSA. From left to right: DAPI (blue) stains nuclear DNA; MCP-tdStayGold (green) marks nuclear MCP signal; and Cy3 (red) visualizes smiFISH targeting the MS2 stem-loops. Transcription sites are highlighted with yellow boxes. Scale bar: 5 µm.

B. The image displays three agarose gels loaded with PCR products targeting the 5' end of the integration sites for two repair cassettes, following amplification of genomic DNA extracts. The expected sizes for the DNA products corresponding to the cassettes containing the blasticidin and hygromycin genes are 1302 bp and 1113 bp, respectively. The H2 clone exhibits only the band for the first CRISPR (HYG), while clones H2-O1 and O1.4 display the expected bands for both repair cassettes (HYG and BSD). A HeLa wild-type DNA extract was used as a negative control, with the specific bands not detected, and H2O served as a PCR negative control. *: non-specific PCR bands. BSD refers to blasticidin and HYG to hygromycin.

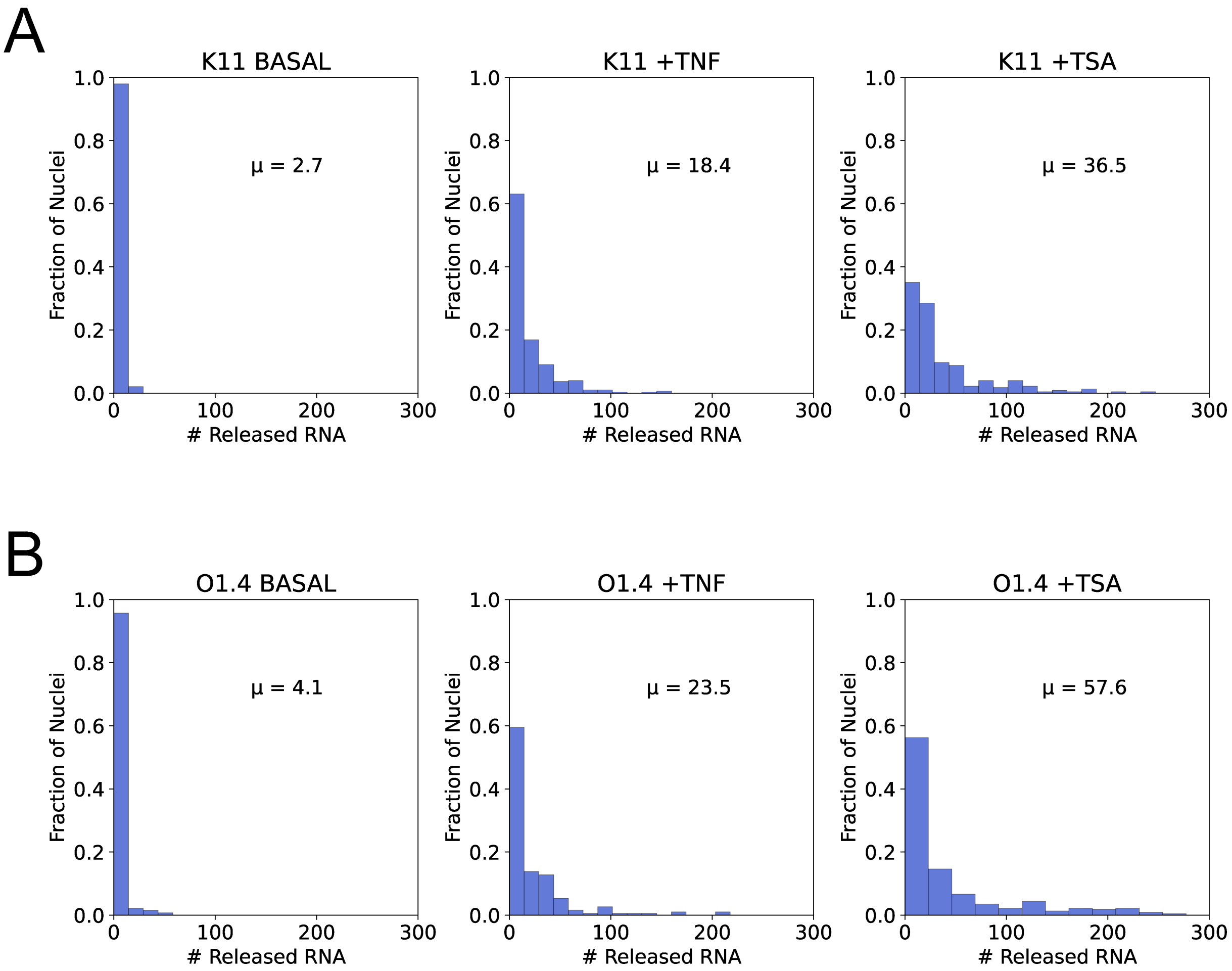

Supplementary Figure 2. Distribution of the number of released RNA molecules per nucleus across conditions in K11 and O1.4 cell lines.

A-B. Distribution of released RNA counts per nucleus for K11 (top row) and O1.4 cells (bottom row), under three conditions: basal, TNFα, and TSA (from left to right). The x-axis represents the number of released RNA molecules per nucleus, while the y-axis indicates the fraction of nuclei with that respective RNA count. The mean RNA count (μ) for each condition is shown in each plot.

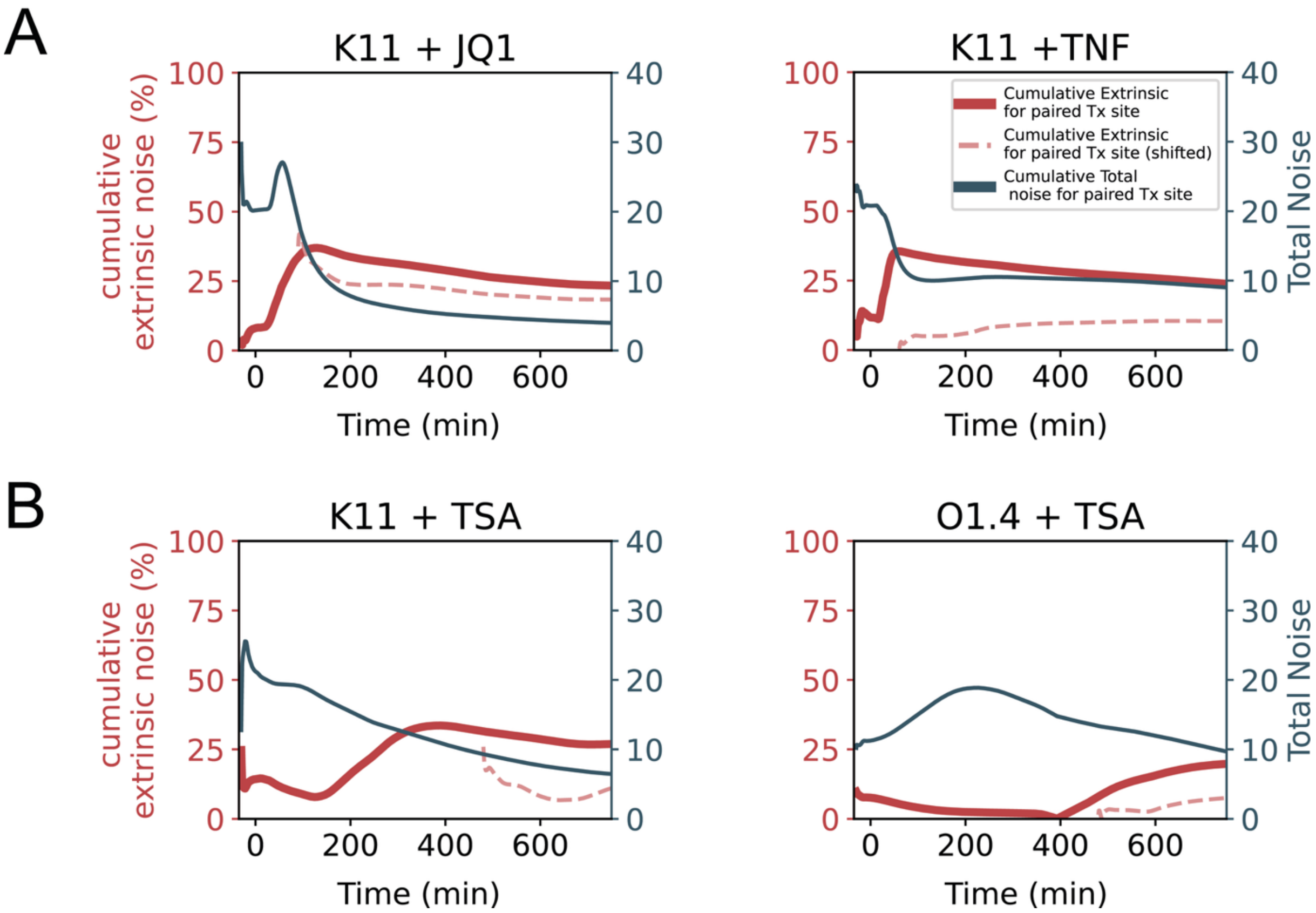

Supplementary Figure 3. Cumulative extrinsic noise analysis over time for clones K11 and O1.4.

A-B. The plots display the cumulative extrinsic noise for clone K11 under TSA and TNF treatment (A), and K11 under JQ1 and O1.4 under TSA (B). The bold red line represents the cumulative extrinsic noise, measured as a percentage of the total noise, while the solid blue line denotes the total noise. The dotted red line shows the cumulative extrinsic noise calculated starting at 60 minutes for TNFα treatment, 480 minutes for TSA treatment and 90 min for JQ1. The left y-axis corresponds to cumulative extrinsic noise in percentage, and the right y-axis represents total noise.

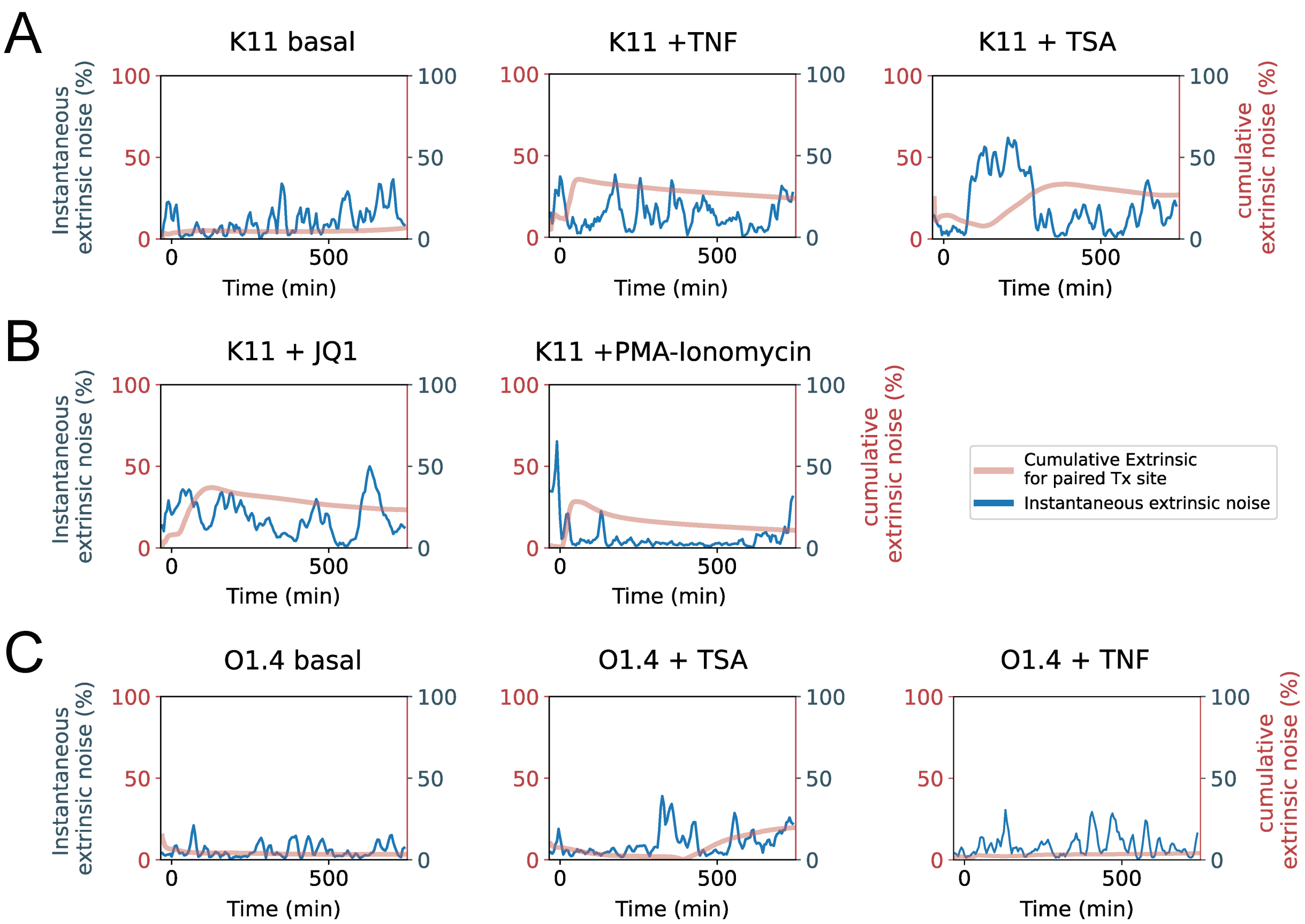

Supplementary Figure 4. Instantaneous vs. cumulative extrinsic noise in clones K11 and O1.4.

A-C. Plots display the cumulative extrinsic noise and instantaneous extrinsic noise for paires transcription sites, for clone K11 (A and B) and O1.4 (C), measured as a percentage of total noise and calculated at each time point under basal condition and after the indicated treatments. The bold red lines represent the cumulative extrinsic noise for paired transcription sites (as a percentage of total noise), while the blue line denotes the instantaneous extrinsic noise for unpaired transcription sites (also measured as a percentage of total noise).

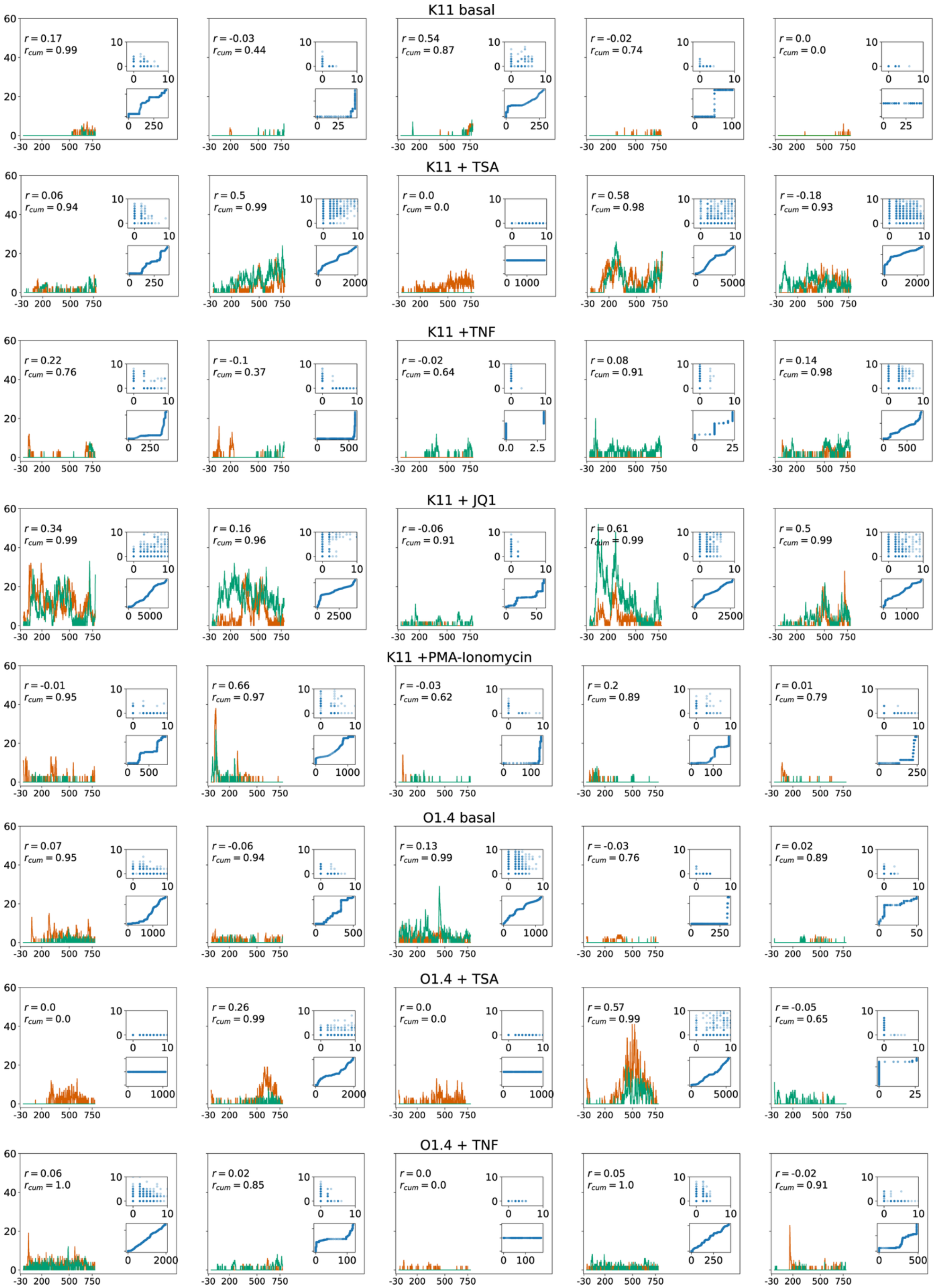
K11 basal
$r = 0.17$ $r_{cum} = 0.99$
$r = -0.03$ $r_{cum} = 0.44$
$r = 0.54$ $r_{cum} = 0.87$
$r = -0.02$ $r_{cum} = 0.74$
$r = 0.0$ $r_{cum} = 0.0$
K11 + TSA
$r = 0.06$ $r_{cum} = 0.94$
$r = 0.5$ $r_{cum} = 0.99$
$r = 0.0$ $r_{cum} = 0.0$
$r = 0.58$ $r_{cum} = 0.98$
$r = -0.18$ $r_{cum} = 0.93$
K11 +TNF
$r = 0.22$ $r_{cum} = 0.76$
$r = -0.1$ $r_{cum} = 0.37$
$r = -0.02$ $r_{cum} = 0.64$
$r = 0.08$ $r_{cum} = 0.91$
$r = 0.14$ $r_{cum} = 0.98$
K11 + JQ1
$r = 0.34$ $r_{cum} = 0.99$
$r = 0.16$ $r_{cum} = 0.96$
$r = -0.06$ $r_{cum} = 0.91$
$r = 0.61$ $r_{cum} = 0.99$
$r = 0.5$ $r_{cum} = 0.99$
K11 +PMA-Ionomycin
$r = -0.01$ $r_{cum} = 0.95$
$r = 0.66$ $r_{cum} = 0.97$
$r = -0.03$ $r_{cum} = 0.62$
$r = 0.2$ $r_{cum} = 0.89$
$r = 0.01$ $r_{cum} = 0.79$
O1.4 basal
$r = 0.07$ $r_{cum} = 0.95$
$r = -0.06$ $r_{cum} = 0.94$
$r = 0.13$ $r_{cum} = 0.99$
$r = -0.03$ $r_{cum} = 0.76$
$r = 0.02$ $r_{cum} = 0.89$
O1.4 + TSA
$r = 0.0$ $r_{cum} = 0.0$
$r = 0.26$ $r_{cum} = 0.99$
$r = 0.0$ $r_{cum} = 0.0$
$r = 0.57$ $r_{cum} = 0.99$
$r = -0.05$ $r_{cum} = 0.65$
O1.4 + TNF
$r = 0.06$ $r_{cum} = 1.0$
$r = 0.02$ $r_{cum} = 0.85$
$r = 0.0$ $r_{cum} = 0.0$
$r = 0.05$ $r_{cum} = 1.0$
$r = -0.02$ $r_{cum} = 0.91$

Supplementary Figure 5. Examples of nascent RNA transcription at dual transcription sites in single cells.

The plots show the nascent RNA trajectories in time for the two transcription sites (red and green) in single cells. The top most inset is a scatter plot of the nascent RNA numbers from the two transcription sites at each time point. Zero nascent RNA means the transcription site is 'OFF'. The bottom inset displays the integrated sum of the nascent RNA numbers at the two transcription sites. The Pearson correlation coefficient of transcription site intensities (r), is displayed at the top left corner of each plot. The Pearson correlation coefficient of the cumulative transcription site intensities (r_{cum}), indicating the linear relationship between the two transcription sites, is displayed at the top-left corner of each plot.

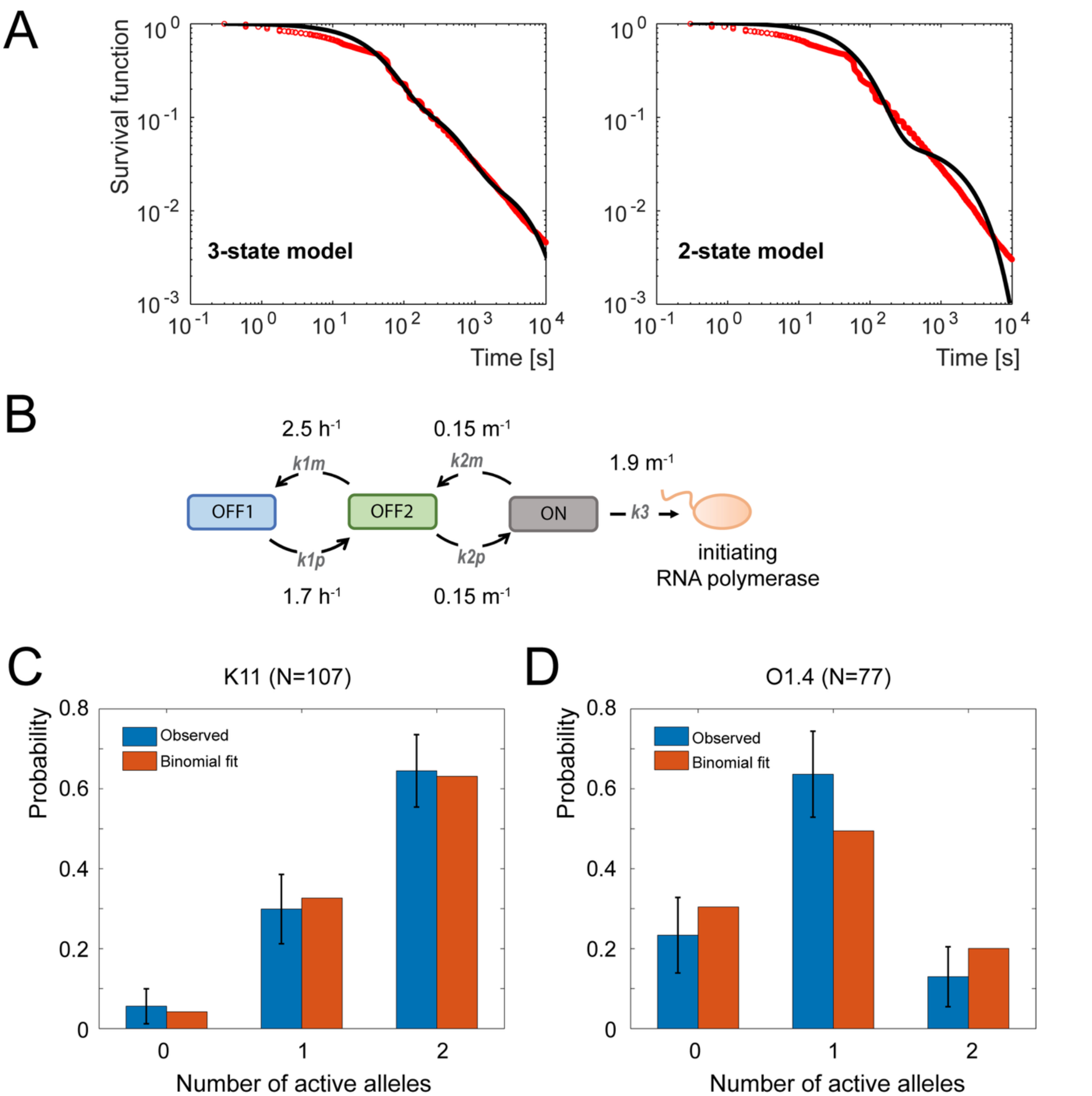

Supplementary Figure 6. Detailed analysis of the O1.4 cells, with and without TNFα treatment.

A. The plot shows the survival function of the waiting times between successive transcription initiation events for O1.4 in the basal state (red), with in black the best fit for a promoter model with three states (left), or two states (without OFF1, right). The 3-state model fits better according to the values of the objective function and the BIC (bayesian information criterium).

B. The rate constant of the best fit 3-state promoter model.

C-D. Plots display the probability of cells to have zero, one or two alleles activated following TNFα stimulation (green), for K11 (C) and O1.4 cells (D). In red, the best fitted binomial distribution. O1.4 cells do not fit well a binomial distribution (p-value 0.01 for a Monte-Carlo goodness of fit). N: number of cells.

List of Supplementary Materials

Supplementary Figures
Supplementary Figure 1: Characterization of the two copy HIV-1 CRISPR clones.
Supplementary Figure 2: Distribution of the number of released RNA molecules per nucleus across conditions in K11 and O1.4 cell lines.
Supplementary Figure 3: Cumulative extrinsic noise analysis over time for clones K11 and O1.4.
Supplementary Figure 4: Instantaneous vs. cumulative extrinsic noise in clones K11 and O1.4.
Supplementary Figure 5: Examples of nascent RNA transcription at dual transcription sites in single cells.
Supplementary Figure 6: Detailed analysis of the O1.4 cells, with and without TNFα treatment.

Supplementary Spreadsheets
Supplementary Spreadsheet 1: RNA count per cell computed from the fixed cell experiments.
Supplementary Spreadsheet 2: RNA count per transcription site computed from the live cell experiments.
Supplementary Spreadsheet 3: MS2 probe sequences used in smFISH experiments.

Supplementary Videos
Video S1: Time-lapse movie showing a cell of the K11 clone under basal conditions, acquired over 10 hours with images captured every minute. A maximum intensity projection is shown.
Video S2: Time-lapse movie showing a cell of the K11 clone treated with 10ng/ml TNF-α, acquired over 10 hours with images captured every minute. A maximum intensity projection is shown.
Video S3: Time-lapse movie showing a cell of the K11 clone treated with 400 nM TSA, acquired over 10 hours with images captured every minute. A maximum intensity projection is shown.
Video S4: Time-lapse movie showing a cell of the K11 clone treated with PMA-Ionomycine, acquired over 10 hours with images captured every minute. A maximum intensity projection is shown.
Video S5: Time-lapse movie showing a cell of the K11 clone treated with 1 µm JQ1, acquired over 10 hours with images captured every minute. A maximum intensity projection is shown.
Video S6: Time-lapse movie showing a cell of the O1.4 clone under basal conditions, acquired over 10 hours with images captured every minute. A maximum intensity projection is shown.
Video S7: Time-lapse movie showing a cell of the O1.4 clone treated with 400 nM TSA, acquired over 10 hours with images captured every minute. A maximum intensity projection is shown.
